# Coincident airway exposure to low-potency allergen and cytomegalovirus sensitizes for allergic airway disease by viral activation of migratory dendritic cells

**DOI:** 10.1371/journal.ppat.1007595

**Published:** 2019-03-07

**Authors:** Sebastian Reuter, Niels A. W. Lemmermann, Joachim Maxeiner, Jürgen Podlech, Hendrik Beckert, Kirsten Freitag, Daniel Teschner, Frederic Ries, Christian Taube, Roland Buhl, Matthias J. Reddehase, Rafaela Holtappels

**Affiliations:** 1 Department of Pulmonary Medicine, University Medical Center Essen-Ruhrlandklinik, Essen, Germany; 2 Institute for Virology and Research Center for Immunotherapy (FZI), University Medical Center of the Johannes Gutenberg-University Mainz, Mainz, Germany; 3 Asthma Core Facility and Research Center for Immunotherapy (FZI), University Medical Center of the Johannes Gutenberg-University Mainz, Mainz, Germany; 4 Department of Hematology, Medical Oncology and Pneumonology, University Medical Center of the Johannes Gutenberg-University Mainz, Mainz, Germany; Thomas Jefferson University, UNITED STATES

## Abstract

Despite a broad cell-type tropism, cytomegalovirus (CMV) is an evidentially pulmonary pathogen. Predilection for the lungs is of medical relevance in immunocompromised recipients of hematopoietic cell transplantation, in whom interstitial CMV pneumonia is a frequent and, if left untreated, fatal clinical manifestation of human CMV infection. A conceivable contribution of CMV to airway diseases of other etiology is an issue that so far attracted little medical attention. As the route of primary CMV infection upon host-to-host transmission in early childhood involves airway mucosa, coincidence of CMV airway infection and exposure to airborne environmental antigens is almost unavoidable. For investigating possible consequences of such a coincidence, we established a mouse model of airway co-exposure to CMV and ovalbumin (OVA) representing a protein antigen of an inherently low allergenic potential. Accordingly, intratracheal OVA exposure alone failed to sensitize for allergic airway disease (AAD) upon OVA aerosol challenge. In contrast, airway infection at the time of OVA sensitization predisposed for AAD that was characterized by airway inflammation, IgE secretion, thickening of airway epithelia, and goblet cell hyperplasia. This AAD histopathology was associated with a T helper type 2 (Th2) transcription profile in the lungs, including IL-4, IL-5, IL-9, and IL-25, known inducers of Th2-driven AAD. These symptoms were all prevented by a pre-challenge depletion of CD4^+^ T cells, but not of CD8^+^ T cells. As to the underlying mechanism, murine CMV activated migratory CD11b^+^ as well as CD103^+^ conventional dendritic cells (cDCs), which have been associated with Th2 cytokine-driven AAD and with antigen cross-presentation, respectively. This resulted in an enhanced OVA uptake and recruitment of the OVA-laden cDCs selectively to the draining tracheal lymph nodes for antigen presentation. We thus propose that CMV, through activation of migratory cDCs in the airway mucosa, can enhance the allergenic potential of otherwise poorly allergenic environmental protein antigens.

## Author summary

From an epidemiological perspective, natural host-to-host transmission of human CMV mostly occurs in early childhood through saliva from virus-shedding intimate contact persons, such as family members or peers. The oronasal route of transmission involves also the airway mucosa and airway-draining lymph nodes. Almost unavoidably, CMV airway infection coincides with airway exposure to environmental antigens, which include potent classical allergens but also protein antigens that have low-to-no allergenic potential on their own. Ovalbumin (OVA), when administered as a purified protein, can serve as a well-studied model antigen for only poorly allergenic environmental antigens. In a murine model of airway exposure to OVA for allergenic sensitization, we have addressed the question if a simultaneous airway infection with murine CMV (mCMV) can promote allergic airway disease (AAD) elicited by challenge exposure to OVA. As anticipated by the model design, exposure to OVA alone did not sensitize for an allergic response to challenge exposure, nor did mCMV infection alone. Notably, based on viral activation of antigen uptake by DCs, both combined sensitized for a type 2 CD4^+^ T helper (Th2) cell-driven AAD histopathology. This is a novel aspect in CMV pathobiology with the medical relevance that CMV airway infection enlarges the spectrum of environmental allergens.

## Introduction

It is common knowledge in the allergy field that viral respiratory infections and allergic airway diseases (AADs), for instance allergic asthma, can interdepend [1,2]. In principle, mutual interference may aggravate or dampen either of these medical entities. While few publications reported on protective effects of viral infections on the development of asthma [3–5], the majority of studies revealed an exacerbation of asthma by viral respiratory diseases (for reviews see [2,6–11]). On the other hand, damage of the asthmatic lung epithelium can increase the susceptibility to viral infections [12]. Allergic asthma and respiratory viral infections both can affect the physical and functional integrity of the airway epithelium and can thereby destroy its barrier function (reviewed in [11]). This in turn facilitates the penetration of allergens as well as the invasion of pathogens [13]. In addition, expression of specific immune response genes in lung epithelial cells can be modulated by allergens and by viruses [14], and there exists evidence to propose a cytokine-based interference between allergic reactions and the antiviral immune response (for an overview see [7]). Overall, the pathophysiological interactions between respiratory virus infections and allergic airway diseases are manifold and rather complicated, and are not yet fully understood.

In addition to the more typical respiratory viruses, such as respiratory syncytial virus (RSV) and rhinoviruses [7] that are characterized by a strict tropism for airway mucosa, cytomegalovirus (CMV), specifically murine CMV (mCMV), was reported to unexpectedly modulate asthma in an experimental mouse model [15,16]. CMVs are strictly host species-specific, double-stranded DNA viruses of the beta-subfamily of the herpesvirus family. Co-speciation with their specific hosts during eons of co-evolution has led to an intricate virus-host adaptation that is reflected by a set of “private” genes for each CMV species that is not shared between CMVs of different host species, but “biological convergence” has led to a comparable pathobiology of different CMV-host pairs [17].

Human CMV (hCMV) is known for its clinical relevance in congenital CMV infection of the fetus, resulting in birth defects, and in the immunocompromised host, in particular in recipients of hematopoietic cell transplantation (HCT) or solid organ transplantation (SOT), in whom it can cause multiple organ failure by infection of a broad range of cell types [18]. This results in tissue-destructive viral histopathology. A link to the airways as a prominent site of CMV pathogenesis is provided by the fact that interstitial CMV pneumonia, associated with infection of interstitial fibroblasts, lung vascular endothelial cells, and pneumocytes, is the lead organ manifestation of hCMV in HCT recipients (for clinical overviews, see [19,20]). The mouse model, employing mCMV, has proven its validity for predictions and “proof of concept” in the immunotherapy of clinical CMV (reviewed in [21–23]). Importantly, all cornerstones of hCMV pathogenesis in HCT patients were reproduced with mCMV in the mouse model of experimental HCT, including interstitial pneumonia [24–29], and lung involvement was also shown in mice infected as neonates [30–32] or as adults [33,34].

In all these instances, CMVs reached the lungs “from within” by intra-host dissemination, and not via the airways. However, although CMVs are not to be viewed as typical respiratory viruses, natural host-to-host transmission through saliva involves infection of airway mucosa, so that airway infection “from without” is epidemiologically relevant. Again, this airway mucosa route of hCMV infection was reproduced in the murine model already in early days of CMV research [35], and revisited more recently [36–38]. Thus, the murine model has proven its validity also with respect to exogenous airway infection.

All in all, there exists reasonable evidence to conclude that respiratory tract infection by host-to-host transmission of CMVs and exposure to inhaled environmental allergens share the target site and thus can meet with the potential consequence of a mutual enhancing and/or inhibiting interference. Clinical observations indeed suggest that CMV infections impact the course of airway diseases of other etiology [39,40], although the underlying mechanisms were at that time not yet fully understood.

Employing the mouse model, the present study aimed at investigating the epidemiologically realistic possibility of sensitization for AAD by airway co-exposure to CMV and an airborne environmental antigen of an otherwise low intrinsic allergenic potential.

## Results

### Model design

Established murine models of AADs, including asthma, are divided into two phases: a sensitization phase, in which the antigen/allergen is usually applied systemically, alone or in combination with an adjuvant, and a challenge phase, in which the antigen/allergen is locally administered to the lungs. At least for studying a potential role of CMV in the sensitization for AAD, this standard protocol with systemic sensitization has no medical correlate and thus required an adaptation to the more realistic scenario of co-exposure to CMV and inhaled environmental antigen in airway mucosa at the time of virus host-to-host transmission.

**Fig 1:**
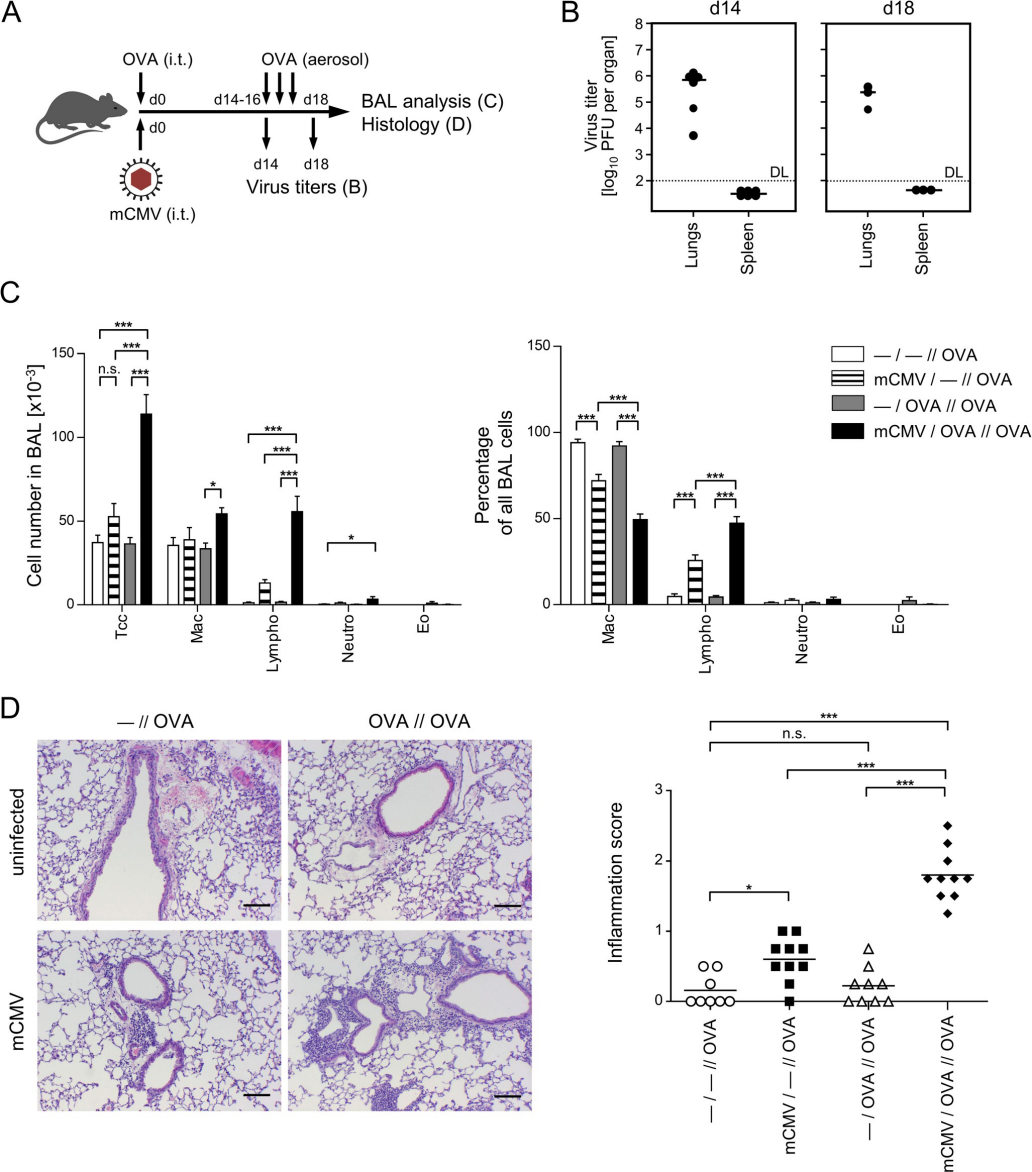
Coincident airway exposure to OVA and mCMV infection predisposes for leucocyte infiltration of the lungs upon OVA aerosol challenge. (A) Experimental design: C57BL/6 mice were i.t. sensitized with purified OVA and infected i.t. with mCMV (day 0), followed by three consecutive airway exposures to nebulized OVA on days 14, 15, and 16. Analyses were performed on day 14, prior to OVA exposure, and on day 18. For experimental groups, see Table 1. (B) Titers of infectious virus in lungs and spleen of group *mCMV/OVA//OVA*. DL, detection limit of 100 PFU per whole organ. Symbols represent individually tested mice. Median values are marked by horizontal bars. (C) Total (left bar diagram) and relative (right bar diagram) number of inflammatory cells present in extravascular lung tissue, determined in BAL fluid. See the internal legend for experimental groups. Tcc, total cell count. Mac, macrophages. Lympho, lymphocytes. Neutro, neutrophilic granulocytes. Eo, eosinophilic granulocytes. Bars represent the mean + SEM of data compiled from 2 independent experiments, each performed with n = 5 mice per experimental group. (D) Left panel: representative histological images of lung tissue obtained by HE staining of lung tissue sections. Bar markers: 100 μm. Right panel: symbols represent the inflammation score for individual mice assessed in a double-blind fashion. Mean values are indicated by horizontal bars. Shown are data from 2 independent experiments with n ≥ 4 mice per experimental group. Comparisons between two groups of interest are indicated by bracket endings. Asterisk-coded statistical significances of the differences: *P≤0.05; ***P≤0.001; n.s.: not significant.

To model this situation, mice were sensitized by intratracheal administration of purified model antigen OVA in absence or presence of a simultaneous intratracheal infection with mCMV, followed two weeks later by three consecutive inhalative challenge exposures to OVA aerosol [41–43]. The experimental regimen is illustrated in Fig 1A. The four experimental groups, on which most conclusions rely, included OVA challenge without prior OVA sensitization, as well as OVA challenge after prior OVA sensitization, both in absence or presence of an mCMV airway infection at the time of OVA sensitization (Table 1).

**Table 1:** Experimental groups.

Group	Sensitization ———/———		Challenge //———
	mCMV	OVA	OVA
—/—//OVA	no	no	yes
mCMV/—//OVA	yes	no	yes
—/OVA//OVA	no	yes	yes
mCMV/OVA//OVA	yes	yes	yes

The sensitization phase is characterized by an uptake of the antigen/allergen by professional antigen-presenting cells (APCs), in particular dendritic cells (DCs), their migration to the draining regional lymph node/s and priming of an adaptive T-cell and B-cell response. Depending on the activation status of the DCs, tolerance or sensitization are induced. Upon successful sensitization, memory cells develop and can react by a recall response to a recurring antigen/allergen exposure. This can result in AAD, which is characterized by an influx of inflammatory cells, such as lymphocytes, macrophages, and granulocytes into the lungs, associated with the release of a large array of inflammatory mediators. Histopathological criteria for AAD are the thickening of airway epithelia and hyperplasia of mucus-producing goblet cells.

### Only airway infection and OVA sensitization combined induce an OVA-specific airway inflammation upon OVA challenge

Inflammatory influx into the airways and lung tissue are signs of AAD. To investigate the influence of mCMV on OVA-specific inflammatory processes in the lungs, immunocompetent C57BL/6 mice were sensitized by intratracheal infection with mCMV combined with intratracheal administration of OVA on day 0. For provoking AAD, repetitive OVA-challenge was performed on days 14, 15, and 16 (Fig 1A). At the time of the first episode of OVA-challenge (day 14) as well as at the time of read-out (day 18), virus replicated locally in the lungs, but not in the spleen (Fig 1B). This organ selectivity is in accordance with findings in a related model of mCMV airway involvement after infection via the intranasal route [36] and is explained by an immune response that prevents further intra-host virus dissemination.

At 48 hrs after the last challenge, cytospin preparations of the broncho-alveolar lavage (BAL) were analyzed for inflammatory cells (Fig 1C), and HE-stained lung sections were scored for an inflammatory cell influx (Fig 1D).

Analyzing the cellular composition of the BAL revealed that only those mice that were mCMV-infected and OVA-sensitized showed elevated overall cell numbers (Fig 1C, left panel) upon OVA challenge (group *mCMV/OVA//OVA*). In particular, numbers of lymphocytes and macrophages were increased in absolute terms. Notably, eosinophilia was not observed and also the number of neutrophilic granulocytes was hardly affected. Airway infection with mCMV in absence of OVA sensitization (group *mCMV/—//OVA*) also induced somewhat higher numbers of lung-infiltrating lymphocytes compared to uninfected groups with either only OVA challenge or OVA sensitization and challenge (groups—*/—//OVA* and—*/OVA//OVA*, respectively), which is explained by OVA-independent, infection-associated and mast cell-assisted lung infiltration [34]. The quantitatively most significant increase in total cell counts (Tcc; Fig 1C, left panel) as well as in the percentage of lymphocytes (Fig 1C, right panel), however, was observed upon OVA challenge when mice were OVA-sensitized in the presence of infection (group *mCMV/OVA//OVA* compared to all other groups). The relative increase in the number of BAL lymphocytes was associated with a relative decrease in the number of alveolar macrophages (Fig 1C, right panel). These findings from cell quantification in the BAL were consistent with corresponding histological images of lung tissue sections, illustrating the most pronounced inflammatory cell influx after OVA challenge in the group of mice sensitized by OVA in the presence of airway infection by mCMV (Fig 1D). Notably, OVA sensitization and challenge in the group—*/OVA//OVA* was not associated with an increased cell infiltration of the lungs, as indicated by an inflammation score that was found to be almost identical to the score in the—*/—//OVA* group of mice with no preceding OVA sensitization (Fig 1D, right panel). In accordance with the cell quantifications, mCMV infection in the OVA-unsensitized control group *mCMV/—//OVA* led to a slightly increased inflammation score but far below the score of the OVA-specific infiltration in the group *mCMV/OVA//OVA*. As all experimental groups included an OVA challenge, it is important to point out that an inflammatory cell influx was not observed in absence of OVA challenge (S1 Fig). Notably, the enhancing effect of mCMV on OVA sensitization was found not to depend on replicative virus (S2 Fig). In conclusion, purified OVA has little-to-no allergenic potential in terms of inducing an inflammatory response, unless sensitization to OVA occurs in the presence of airway exposure to mCMV.

### Only airway infection and OVA sensitization combined prime for an OVA-specific antibody recall response

**Fig 2:**
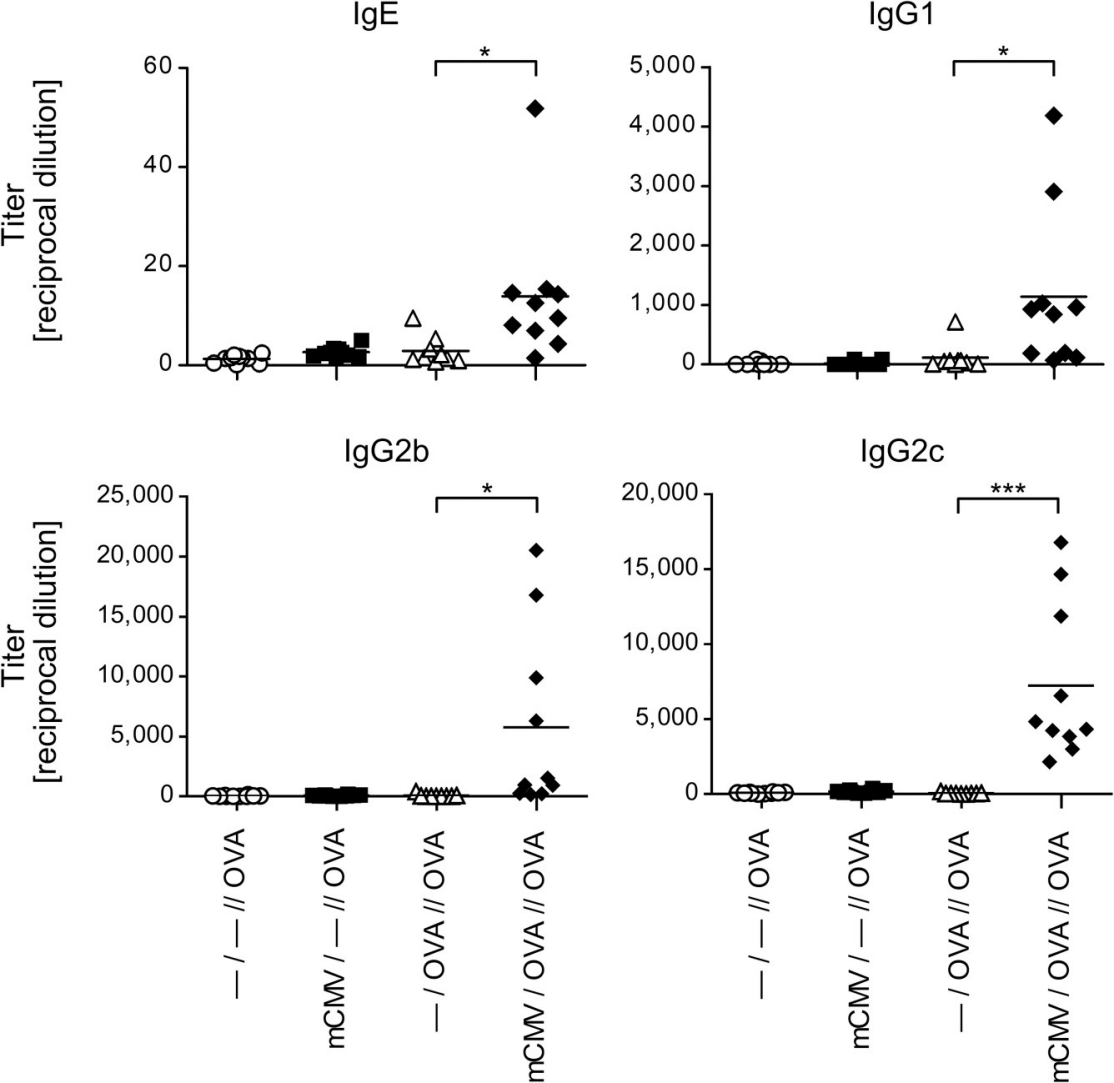
Impact of mCMV infection on the production of OVA-specific immunoglobulins. Experimental design as outlined and explained in Fig 1A and Table 1. Sera were recovered at 48 hrs after the last challenge exposure to OVA aerosol, and were analyzed for the titers of OVA-specific antibodies of the classes IgE, IgG1, IgG2b, and IgG2c. Symbols represent data from individual mice compiled from 2 independent experiments, each performed with n = 5 mice per experimental group. Mean values are indicated by horizontal bars. Asterisk-coded statistical significance: *P≤0.05; ***P≤0.001.

As mCMV airway infection in the OVA sensitization phase was found to enhance an OVA-specific airway inflammation upon OVA challenge (see above), we also analyzed the influence of mCMV infection on the OVA-specific B-cell response (Fig 2). To this end, OVA-specific serum immunoglobulins were measured at 48 hrs after the last challenge. Intriguingly, OVA sensitization and challenge in experimental group—*/OVA//OVA* failed to induce OVA-specific IgE, IgG1, IgG2b and IgG2c antibodies, neither did mCMV airway infection in absence of OVA sensitization. Again, only a combination of mCMV airway infection with OVA sensitization and challenge in group *mCMV/OVA//OVA* resulted in significant titers of OVA-specific antibodies. Importantly, as antibody production and immunoglobulin class switch are CD4^+^ T helper cell-dependent, these results imply that sufficient help was provided only when CD4^+^ T cells were primed by OVA sensitization under conditions of concomitant infection.

### Only airway infection and OVA sensitization combined induce an OVA-specific histopathology characteristic of AAD

**Fig 3:**
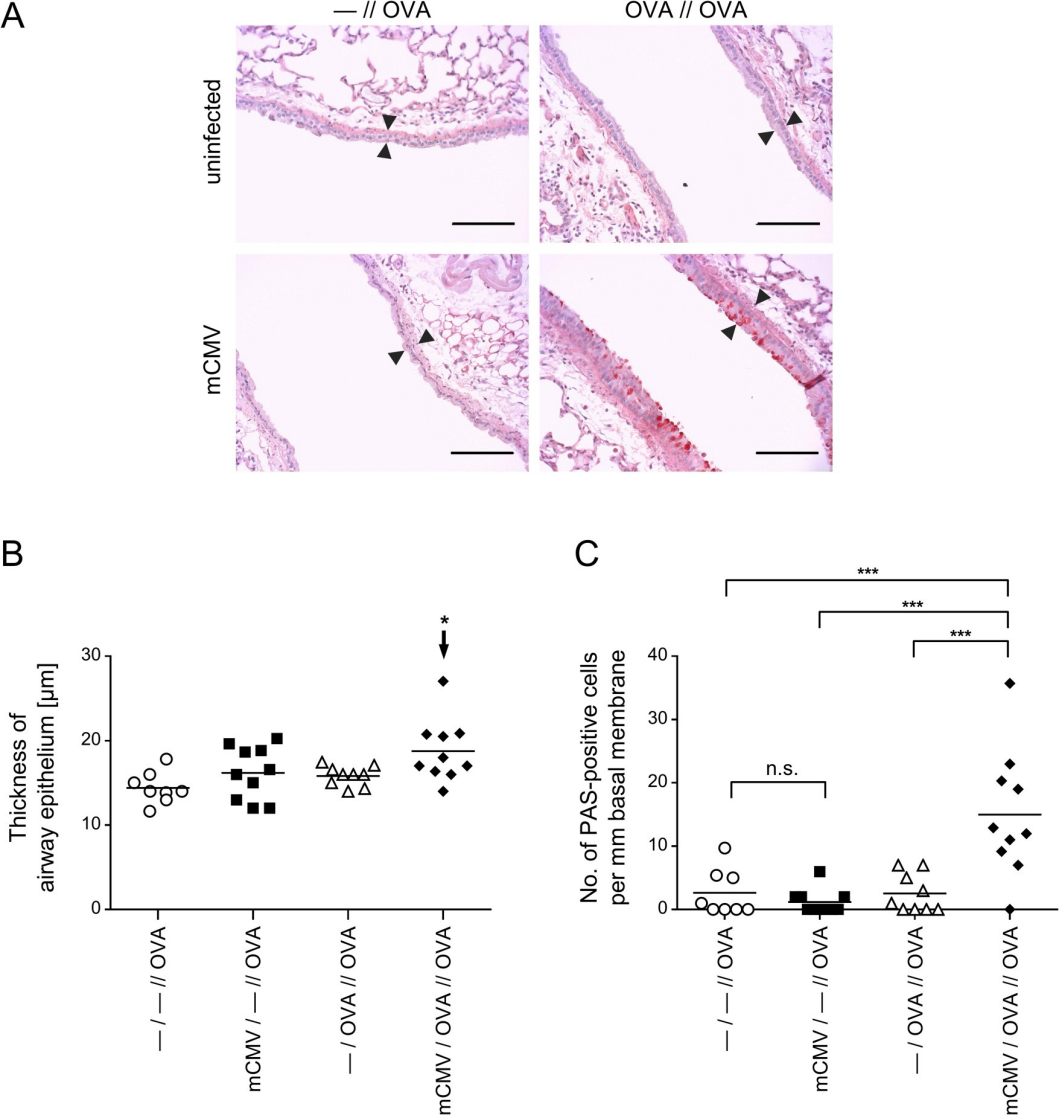
Impact of mCMV infection on OVA-specific airway histopathology. Experimental design and groups as outlined and explained in Fig 1A and Table 1. (A) Histological images of lung tissue remodeling in response to OVA aerosol challenges. Lung tissue sections were PAS/HE stained to visualize and count mucus-producing, PAS-positive goblet cells. Shown are images of representative sections. Bar markers: 100 μm. Opposed arrowheads highlight differences in the thickness of airway epithelia. (B) and (C) Diagrams of thickness of airway epithelia and number of mucus-producing, PAS-stained goblet cells, respectively. Symbols represent data from individual mice, corresponding to the mice of Fig 1D. Mean values are indicated by horizontal bars. The arrow in (B) points to the experimental group that was identified by ANOVA as being significantly different in the comparison of all 4 groups. Asterisk-coded statistical significances: *P≤0.05; ***P≤0.001; n.s.: not significant.

Remodeling of the airways by increased numbers of mucus-secreting goblet cells, that is goblet cell hyperplasia, represents a histopathological hallmark defining AAD more stringently than inflammatory cell influx alone, in particular when studied in the presence of infection that by itself contributes to inflammation. Histological images of lung tissue sections document thickening of the bronchial epithelium and enhanced numbers of PAS-stained, mucus-producing goblet cells upon OVA challenge only when OVA sensitization had taken place in the presence of mCMV airway infection (Fig 3A, lower right panel). This visual impression, documented by representatively selected images of tissue sections, is statistically substantiated by histometrical quantitation of the thickness of airway epithelia (Fig 3B) and by counting of goblet cells (Fig 3C). It is of interest to note that the comparison between control group—*/—//OVA* and infected group *mCMV/—//OVA* did not reveal a significant difference in these parameters of AAD (Fig 3B and 3C). Thus, whereas mCMV infection by itself is associated with cellular infiltration of the lungs (see above), it does not elicit goblet cell hyperplasia, a finding that is most important as it clearly distinguishes viral histopathology in the lungs from OVA-specific AAD.

### Intratracheal infection preferentially recruits CD8^+^ effector T cells to the lungs

**Fig 4:**
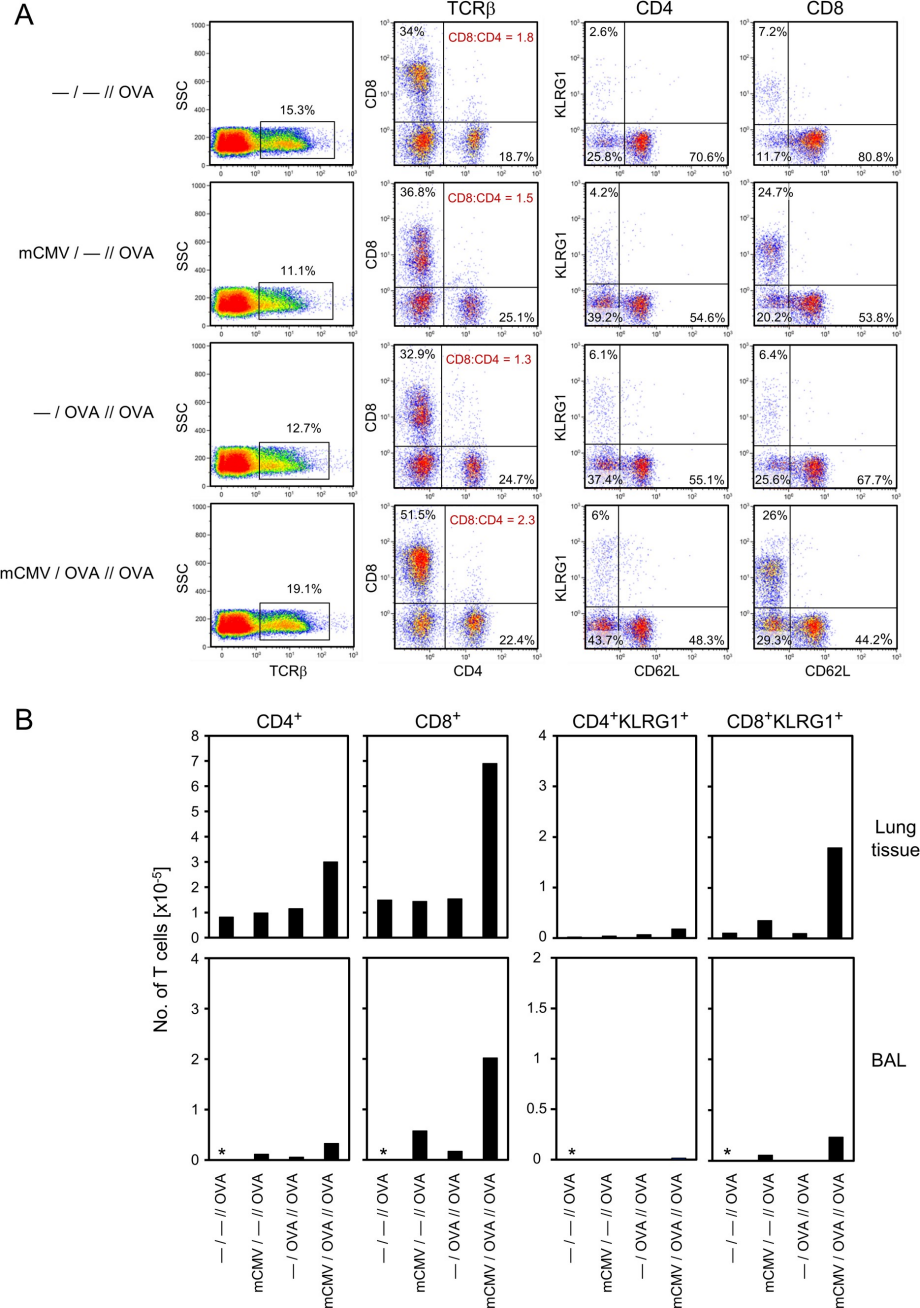
Cell surface phenotype of lung-infiltrating T-cell subpopulations. Experimental design as outlined and explained in Fig 1A and Table 1. At 48 hrs after the last challenge exposure to OVA aerosol, cells were recovered from the lungs by dissociation of lung tissue or by BAL. Yields from 5 mice per experimental group were pooled for cytofluorometric analysis. (A) T-cell subpopulations and their activation phenotypes among cells recovered from lung tissue are shown in dot plots for the indicated combinations of markers after electronic gating on T cells defined by the expression of the TCRβ chain. Percentages of interest are indicated in the quadrants that were defined by isotype controls. For the corresponding analysis of BAL, see S3 Fig. (B) Absolute yields per lung of the indicated T-cell phenotypes calculated from the cytofluorometrically determined proportions and the absolute counts of live cells recovered from digested lung tissue or by BAL from 5 mice pooled. *, cell yield from BAL too low for reliable quantitation.

Since CD8^+^ T cells are long known as the predominant effector cell type that controls acute pulmonary mCMV infection in related experimental mouse models ([24,26,27,28,34], reviewed in [23,44]), it was an obvious question if CD8^+^ T cells with an effector cell phenotype dominate T-cell infiltrates in the lungs also in the here discussed AAD model (Fig 4).

At a glance, CD8^+^ T cells quantitatively dominated over CD4^+^ T cells in pulmonary T-cell infiltrates in overall lung tissue (Fig 4A and 4B) and in BAL that represents cells present in extravasal airway epithelia including alveolar epithelium of the lungs (Fig 4B; S3 Fig). The highest percentage and absolute number of CD8^+^ T cells and, accordingly, the highest CD8:CD4 T-cell ratio, was observed in the group *mCMV/OVA//OVA*. A direct comparison of this group with the likewise infected group *mCMV/—//OVA*, not sensitized to OVA, suggests an OVA sensitization-specific component of the response, which is more evident in lung tissue than it is in BAL. In the CD8^+^ T-cell population, numbers of CD62L^-^KLRG1^+^ short-lived effector cells (SLECs) [45–48], which include terminally differentiated cells destined to death [49], were elevated in both infected groups compared to uninfected groups in lungs and BAL. A slightly elevated number of CD8^+^ SLECs in the infected and OVA-sensitized group *mCMV/OVA//OVA* may indicate a minor OVA-specific component in an overall primarily virus-specific CD8^+^ SLEC population. Percentages of CD4^+^ SLECs were low throughout and were not notably elevated in the infected groups, neither in lungs nor in BAL (Fig 4A and 4B; S3 Fig), although slightly elevated absolute numbers in the group *mCMV/OVA//OVA* (Fig 4B) might indicate a minor OVA sensitization-specific component as well. Pulmonary infiltrates in all groups included an unexpectedly high proportion of TCRβ^+^CD4^-^CD8^-^ cells amongst all TCRβ^+^ cells, likely representing NKT cells [50,51]. As their proportion was similar in all groups regardless of infection or OVA sensitization, and as their presence after challenge evidently did not correlate at all with AAD (recall Fig 3), we decided not to pursue this otherwise interesting phenomenon further in the context of the here discussed AAD model.

**Fig 5:**
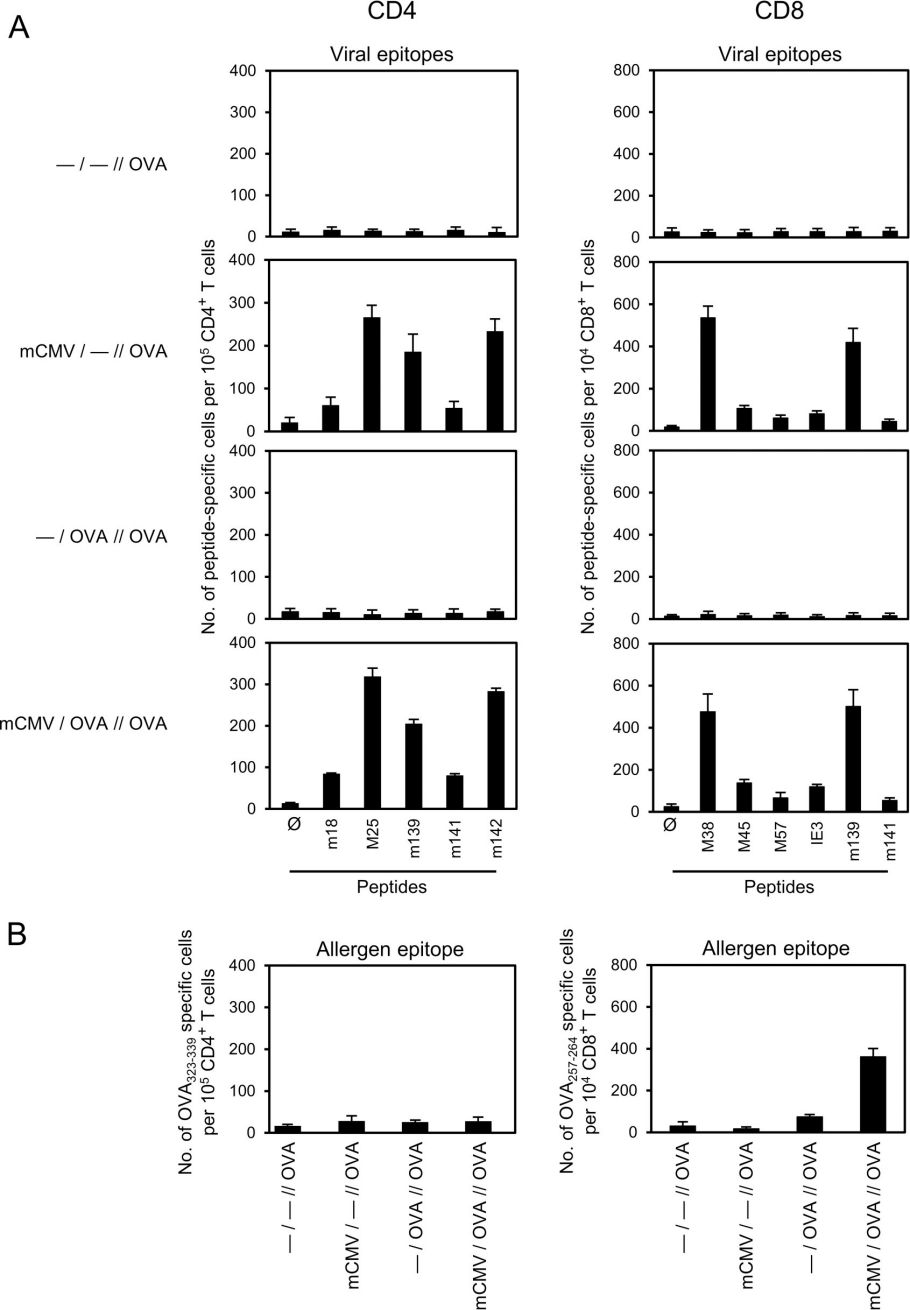
Epitope-specificity repertoire of CD4^+^ and CD8^+^ T cells in the lungs. For experimental design and codes of experimental groups, see Fig 1A and Table 1. Frequencies of epitope-specific cells among immunomagnetically-purified CD4^+^ and CD8^+^ T cells, retrieved from lung tissue at 48 hrs after the last challenge exposure to OVA aerosol, were determined by IFN-γ-based ELISPOT assay after sensitization with synthetic antigenic peptides. (A) Frequencies of CD4^+^ and CD8^+^ T cells recognizing the indicated viral epitopes. (B) Frequencies of CD4^+^ and CD8^+^ T cells recognizing the OVA epitopes OVA_323-339_ (ISQAVHAAHAEINEAGR) and OVA_257-264_ (SIINFEKL), respectively. Bars represent most probable numbers calculated by intercept-free linear regression analysis of data from graded numbers of effector cells each tested in triplicate cultures. Error bars indicate the 95% confidence intervals.

Despite the low numbers of CD4^+^ SLECs, viral epitope-specific cells capable of secreting IFN-γ upon short-term stimulation with a panel of known antigenic peptides of mCMV presented by the MHC class-II (MHCII) molecule I-A^b^ [52,53] were detectable in the two infected groups (Fig 5A, left panel), including group *mCMV/—//OVA* in which AAD did not develop. So, virus-specific IFN-γ-secreting CD4^+^ T cells are apparently not notably involved in AAD. Surprisingly, only baseline levels of CD4^+^ T cells recognized an immunodominant I-A^b^-presented OVA peptide [54], with no enhancement observed in the AAD group *mCMV/OVA//OVA* (Fig 5B, left panel). As IFN-γ is a lead cytokine of T helper type-1 (Th1) cells, we also quantitated CD4^+^ T cells expressing the lead cytokine IL-4 of Th2 cells after short-term stimulation with the I-A^b^-presented peptides. Although cells secreting IL-4 were easily detectable in the AAD group *mCMV/OVA//OVA* after polyclonal stimulation via antibody ligation of the signaling molecule CD3ε, numbers of Th2 cells specific for the tested panel of viral peptides as well as the OVA peptide were baseline throughout (S4 Fig). Thus, viral epitope-specific CD4^+^ T cells in the infected groups were almost exclusively Th1 cells, whereas the frequencies of OVA-specific Th1 and Th2 cells were all below the detection limit of this assay.

As expected, IFN-γ-secreting CD8^+^ T cells specific for a panel of known antigenic peptides of mCMV presented by the MHC class-I (MHCI) molecules K^b^ and D^b^ [55] were detected in the two infected groups (Fig 5A, right panel). As with the virus-specific CD4^+^ T cells discussed above, absence of AAD in group *mCMV/—//OVA* implies that virus-specific CD8^+^ T cells are also not critically involved in AAD. At first glance, absence of viral epitope-specific CD8^+^ T cells in the OVA-sensitized but uninfected groups may seem to be trivial, but it actually gives us the important information that there exists no antigenic mimicry between the K^b^-presented immunodominant OVA epitope SIINFEKL and any of the K^b^- or D^b^-presented viral epitopes that could lead to cross-reactivity of CD8^+^ T cells defining heterologous immunity [56,57]. A low frequency of SIINFEKL-specific CD8^+^ T cells was detected in group*—/OVA//OVA*, and significantly enhanced in the AAD group *mCMV/OVA//OVA* (Fig 5B, right panel). As antigen processing of exogenously administered OVA and presentation of OVA-derived antigenic peptide by an MHCI molecule involves OVA uptake and cross-presentation by DCs (for reviews, see [58,59]), the enhanced SIINFEKL-specific response in group *mCMV/OVA//OVA* was the first indication for viral activation of antigen cross-presenting DCs in this AAD model.

Altogether, at this stage of investigation, it was tempting to conclude that CD4^+^ T cells are unlikely to contribute decisively to AAD observed in group *mCMV/OVA//OVA*, whereas an OVA-specific CD8^+^ T-cell response appeared to correlate with AAD.

### OVA-specific AAD is caused by CD4^+^ T cells

**Fig 6:**
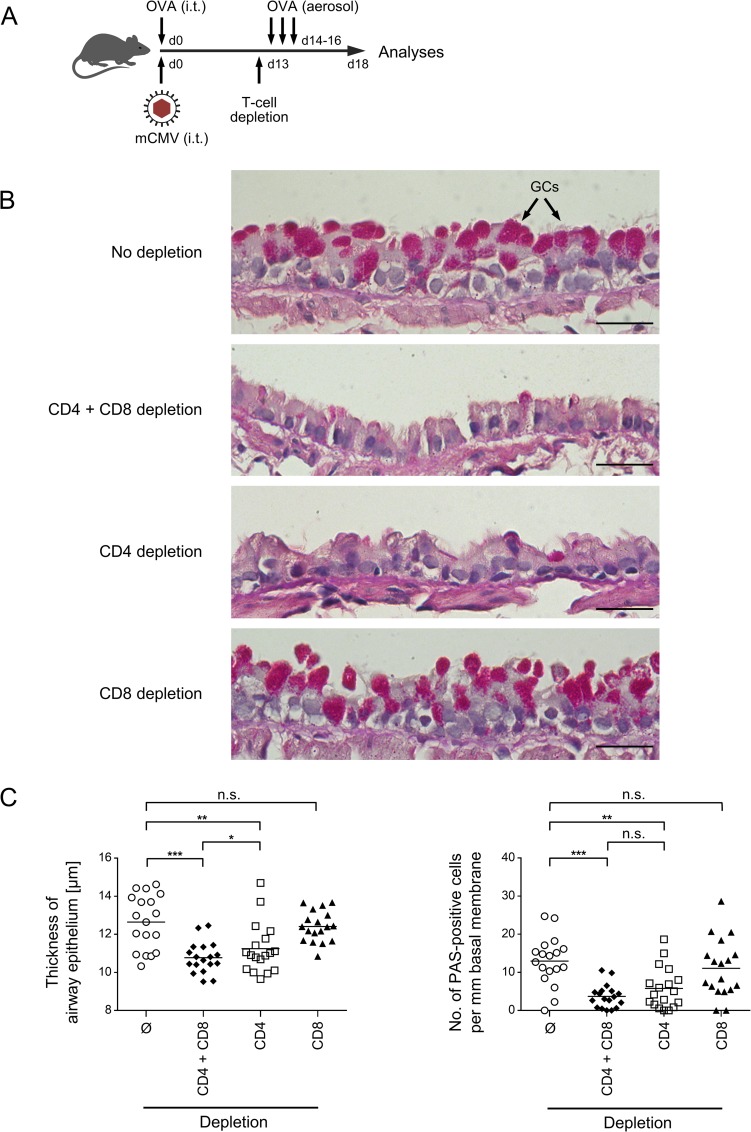
Impact of CD4^+^ and CD8^+^ T cells on OVA-specific airway histopathology. (A) Experimental design: C57BL/6 mice were i.t. sensitized with purified OVA and infected i.t. with mCMV (day 0), followed by three consecutive airway challenges with aerolized OVA on days 14, 15, and 16. One day before the first challenge (d13), CD4^+^ T cells, or CD8^+^ T cells, or both were depleted. Analyses were performed on day 18. (B) Histological images of lung tissue-remodeling in response to OVA challenge and modulated by preceding T cell-subset depletions. Lung tissue sections were PAS/HE stained to visualize and count mucus-producing, PAS-positive goblet cells in bronchial epithelium. Shown are images of representative sections. Bar markers: 25 μm. GCs, goblet cells. (C) Diagrams of the thickness of airway epithelia and the number of mucus-producing, PAS-stained goblet cells, respectively. Symbols represent data from randomly selected 18 tissue sections derived from the lungs of 6 individual mice per group to account for both inter-individual variance and intra-tissue variance. Mean values are indicated by horizontal bars. Asterisk-coded statistical significances: *P≤0.05; **P≤0.01; ***P≤0.001; n.s.: not significant.

To unequivocally identify the effector cells in AAD, mice of the AAD group *mCMV/OVA//OVA* were either left untreated to result in AAD, or were depleted of T-cell subsets in all combinations shortly before the first OVA challenge (Fig 6A). The result was as clear as surprising: goblet cell hyperplasia characterizing AAD in the undepleted mice (Fig 6B, upper image) was prevented by double depletion as well as by CD4 depletion alone, but not by CD8 depletion alone (Fig 6B). These findings, visualized by representatively selected histopathological images, were statistically substantiated by measuring the thickness of airway epithelium and by counting the number of goblet cells (Fig 6C). For both parameters of AAD, a significant reduction was achieved by double depletion and by CD4 depletion, but not by CD8 depletion alone. A trend to a slightly more efficient reduction by depletion of both T-cell subsets, as compared to CD4 depletion alone, did not reach statistical significance for goblet cell hyperplasia. Thus, although CD8^+^ T cells by far dominate the T-cell response in quantity, AAD histopathology is caused by the minority population of CD4^+^ T cells.

### AAD correlates with a Th2 cytokine transcription profile in the lungs

In other AAD models, specifically in murine asthma, airway goblet cell hyperplasia was found to be directly induced by Th2-derived IL-9, whereas IL-4, IL-5, and IL-13 independently induce goblet cell hyperplasia, though indirectly via neutrophilic granulocytes [60–62]. IL-25 has also been implicated in AAD, operating indirectly by stimulating the production of IL-4, IL-5, and IL-13 (reviewed in [63]).

Commemorating the unverifiably low frequency of IL-4-secreting peptide-specific Th2 cells in the lungs of mice of the relevant AAD group *mCMV/OVA//OVA* (S4 Fig), we decided to perform a cytokine transcription profiling in the lungs, as it is less dependent on the time point of analysis, is more sensitive because of amplification by RT-PCR, and covers more candidates than a protein array. Specifically, cytokines with a short half-life delivered by very few producer cells over a short distance to target cells in tissue are difficult to detect as proteins *in situ*. Furthermore, at least for T cells there exist no example and no rationale to give reasons for a speculation that gene expression would not lead to the respective cytokine.

**Fig 7:**
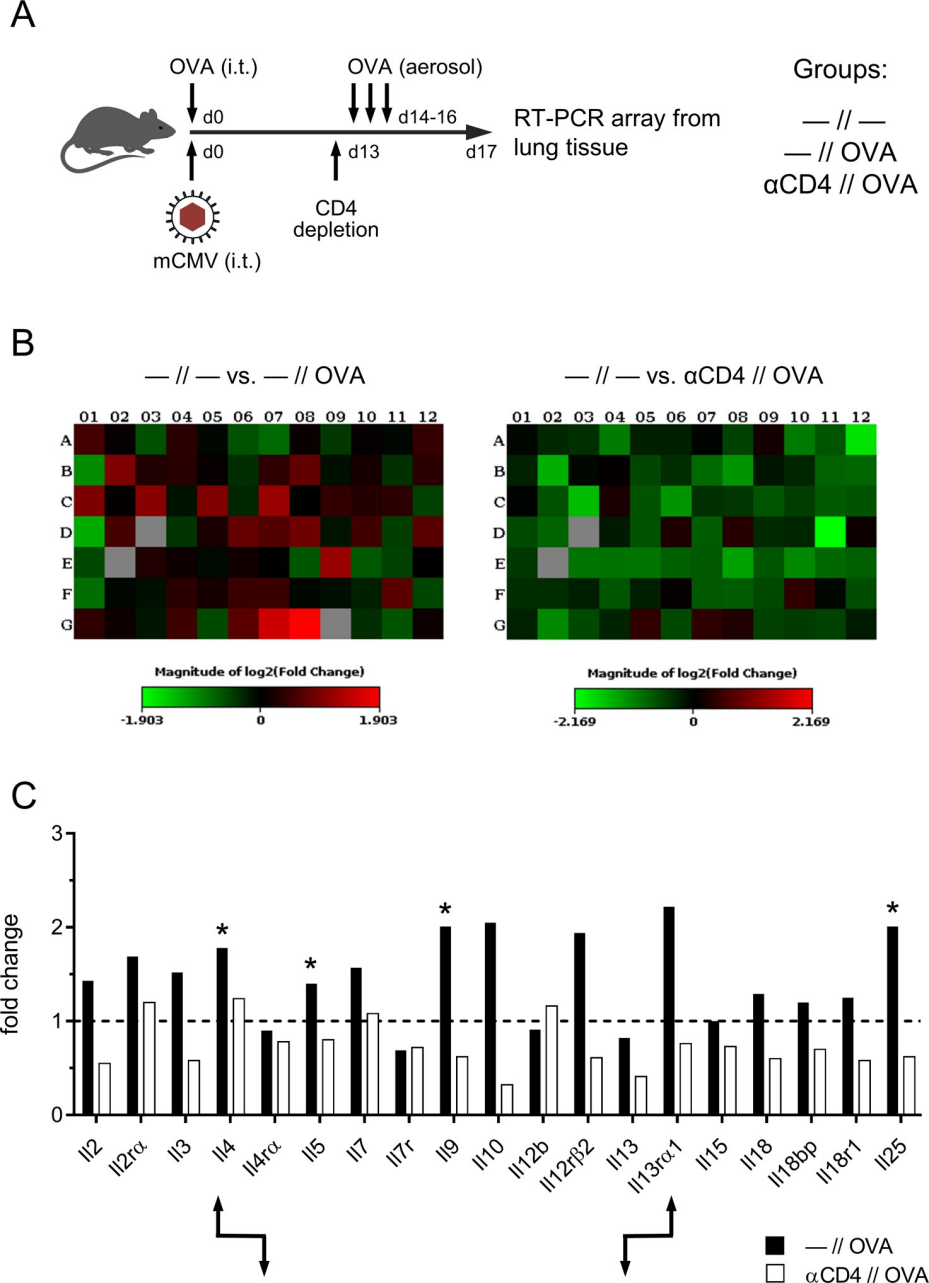
Th2 cytokine expression profile in AAD lungs. (A) Experimental design: all C57BL/6 mice in the three experimental groups of this experiment were mCMV/OVA-sensitized by airway co-exposure on day 0. Group*—//—*was not treated further. Group*—//OVA*, that is the AAD group, received a challenge by three consecutive airway exposures to aerolized OVA on days 14, 15, and 16. Group αCD4//OVA was depleted of CD4^+^ T cells on day 13, that is one day before starting the three consecutive challenge exposures. Mouse Th1&Th2-response RT-PCR arrays were performed with purified lung RNA preparations on day 17. (B) Heat maps of changes in gene expression levels induced by OVA challenge in the presence of CD4^+^ T cells (left panel) or after depletion of CD4^+^ T cells (right panel), both relative to no OVA challenge. (C) Comparisons of expression levels for selected genes of interest. The bar diagram shows fold-changes, calculated by using the ΔΔCt method. The baseline expression levels defined by group*—//—*were set as value 1 (dashed line). Asterisks mark cytokines known to be involved in Th2-driven AAD. Arrow symbols point to examples of inverse gene expression regulation for cytokines and their corresponding receptors.

A comparison of transcriptional activity in lungs of mCMV/OVA-sensitized mice with no OVA challenge (no AAD) and after OVA challenge (AAD) revealed a broad challenge-dependent induction of cytokine gene expression with a predominantly Th2 profile (Fig 7; S5 Fig), which includes the known AAD/goblet cell hyperplasia-inducing cytokines IL-4, IL-5, IL-9, and IL-25, with the notable exception of IL-13. The source of most of the induced cytokines was clearly CD4^+^ T cells, as CD4 depletion prior to OVA challenge often not only precluded an induction but even led to a reduction to below the pre-challenge level. Notably, cytokine-receptor pairs showed the known inverse regulation in that upregulation of the cytokine leads to downregulation of its receptor (as an example, see IL-4/IL4rα) and downregulation of the cytokine leads to upregulation of its receptor (as an example, see IL-13/IL13rα1). This is a further argument to conclude that the respective cytokines were indeed produced in the lungs.

**Fig 8:**
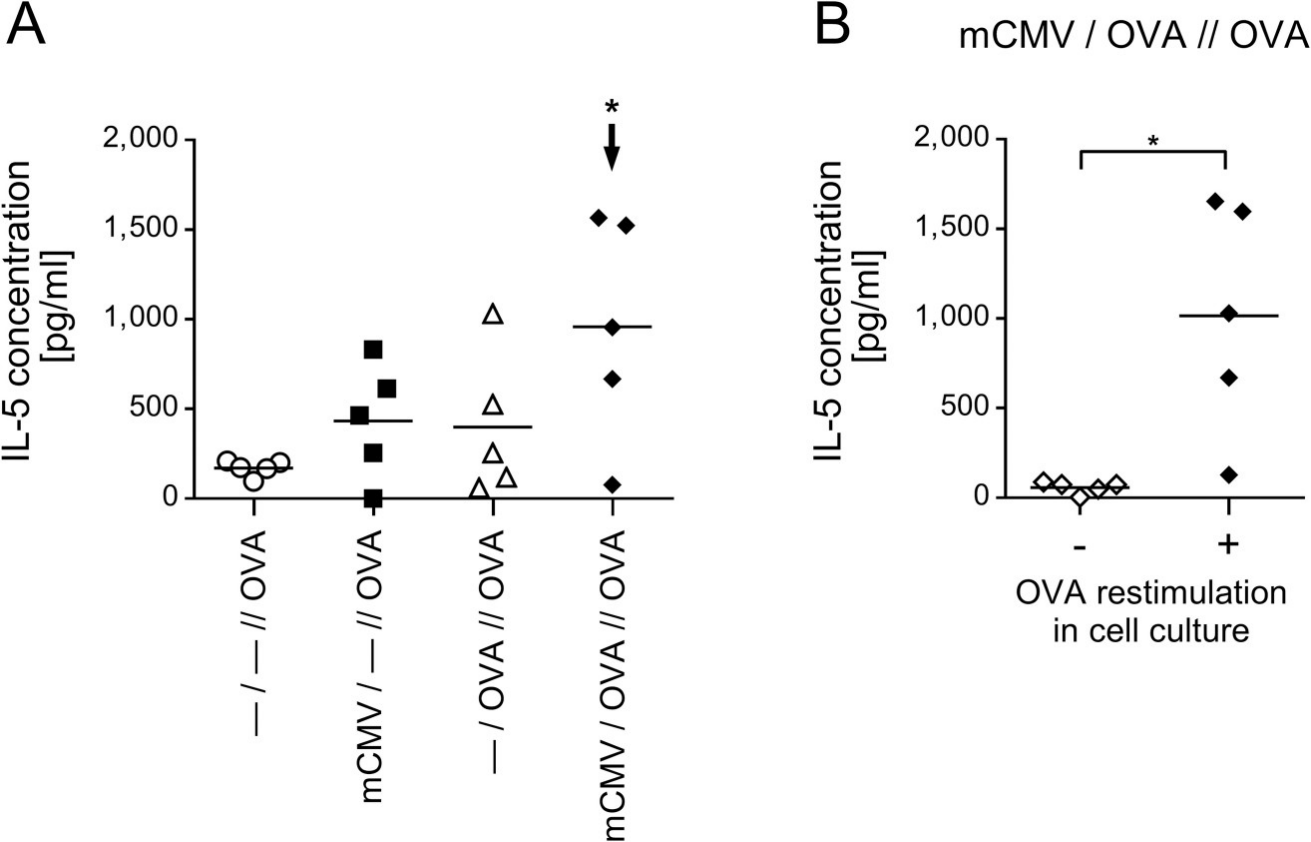
Enhanced production of AAD-driving Th2 lymphokine IL-5 in OVA-restimulated cells derived from AAD lungs. Experimental design as outlined in Fig 1A, with the four experimental groups listed in Table 1. Lung cells isolated on day 18 were restimulated in cell culture with OVA for 72 hrs, and IL-5 was measured in the culture supernatants. (A) Values represent the difference in IL-5 levels between test cultures with OVA restimulation and unstimulated control cultures, thus representing the OVA-specific IL-5 induction. (B) Values represent IL-5 concentrations in cell cultures of the AAD group either restimulated with OVA or left without restimulation. Throughout, symbols represent data from lung cell cultures corresponding to individual mice tested. The median values are indicated. The arrow in panel A indicates the experimental group that was identified by ANOVA as being significantly different in the comparison between all 4 groups with P≤0.05 (*). Significance with P≤0.05(*) in panel B is based on comparison between the two groups by using the unpaired two-tailed Student’s t test with Welch’s correction of unequal variances.

Enhanced presence of Th2 cells in AAD lungs was finally confirmed by amplifying IL-5 production in lung cells, derived from lungs of all four experimental groups (Table 1), by restimulation with full-length OVA protein in cell culture, which covers all epitopes. Notably, OVA-specific IL-5 production, measured as difference between OVA restimulated cultures and non-restimulated control cultures, was significantly enhanced in the AAD group *mCMV/OVA//OVA* as compared to the remaining three groups in which AAD did not develop (Fig 8A). Absolute IL-5 levels in cell cultures of lung cells of the relevant AAD group *mCMV/OVA//OVA* demonstrate the OVA-specificity of this recall response (Fig 8B). This finding finally proved the presence of OVA-specific, IL-5-producing Th2 cells in the AAD lungs.

### Airway infection activates DCs and enhances OVA uptake in the sensitization phase

**Fig 9:**
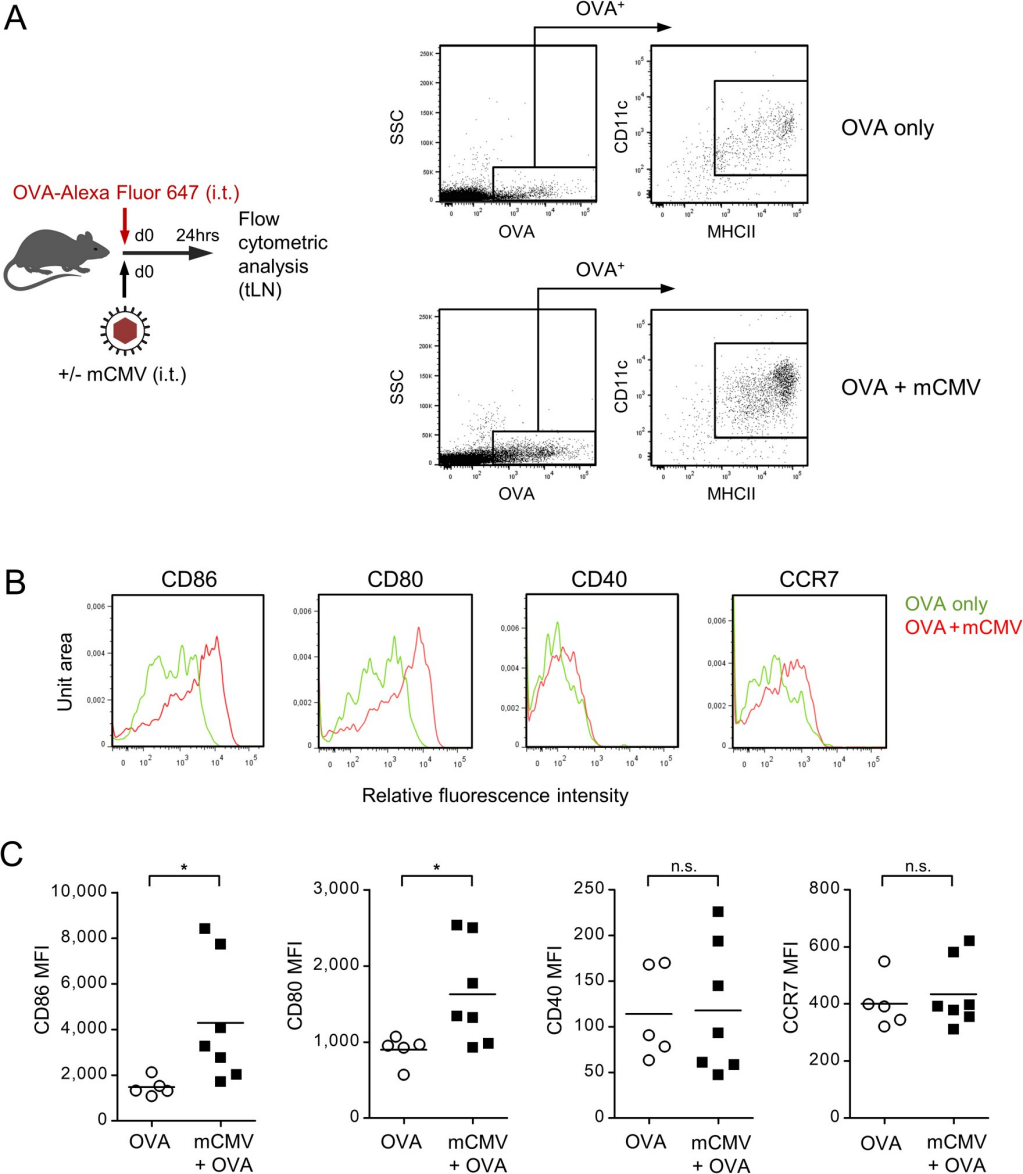
Respiratory mCMV infection activates DCs for antigen uptake and migration. (A) Experimental design and gating strategy: To assess antigen uptake by DCs and to track the DCs, C57BL/6 mice were sensitized by airway exposure to fluorescent OVA-Alexa Fluor 647 on day 0 in absence (OVA only) or presence of airway co-exposure to 10^6^ PFU of mCMV (OVA + mCMV). Cytofluorometric analysis was performed 24 hrs later with cells isolated from the tracheal lymph nodes (tLN) that drain the tracheal mucosa. Doublets were excluded from the analysis and an electronic gate was set on cells that had taken up the labeled OVA. Cells in this gate were identified as DCs based on cell surface expression of CD11c and MHCII. SSC, sideward scatter. (B) Cell surface expression of the indicated activation markers expressed by OVA-laden CD11c^+^MHCII^+^ DCs shown in representative fluorescence histograms. (C) Diagrams of mean fluorescence intensities (MFI), with symbols representing data for mice tested individually. Mean values are indicated by horizontal bars. Shown are data compiled from 2 independent experiments. Asterisk-coded statistical significance: *P≤0.05; n.s.: not significant.

The question remained how mCMV airway infection facilitates OVA sensitization of Th2 cells. Sensitization by an airborne environmental antigen requires antigen uptake by activated DCs in the airway mucosa, migration of antigen-laden DCs to draining regional lymph nodes, specifically the tracheal lymph nodes (tLN), and antigen presentation to naïve Th2 cells. To track the antigen, mice were sensitized with a fluorescent (Alexa Fluor 647) derivate of OVA, administered intratracheally alone or in combination with mCMV (Fig 9A). As soon as 1 day later, labeled OVA had reached the tLN, where it localized almost exclusively to MHCII^+^CD11c^+^ DCs. Notably, the number of OVA-laden DCs in the tLN was markedly elevated after airway co-exposure to mCMV, so that one role of mCMV apparently is to facilitate antigen uptake by DCs. More efficient uptake of labeled OVA was associated with activation of DCs (Fig 9B and 9C), as reflected by a significant upregulation of co-stimulatory molecules, such as the CD28 ligands CD80 (B7-1) and CD86 (B7-2) [64], which are components of the immunological synapse critical for the priming of naïve T cells [65,66]. In contrast, cell surface expression of CD40 and the chemokine receptor CCR7/CD197 on OVA-laden DCs was not enhanced by airway co-exposure to mCMV.

### Airway infection with mCMV promotes the migration of OVA-laden CD11b^+^ and CD103^+^ cDCs selectively into the draining tLN

So far, data have shown the presence of activated, OVA-laden MHCII^+^CD11c^+^ DCs in the airway-draining tLN after combined airway exposure to OVA and mCMV. A variety of distinct DC subsets localize to the respiratory tract, where they survey the respiratory mucosa and parenchyma for foreign antigens, including pathogens. Upon receiving an activation stimulus and uptake of antigen/allergen, several subsets of airway-resident DCs are able to migrate to the draining tLNs, where they process and present antigens for sensitization of naïve T cells or for restimulation of memory T cells [67–69].

**Fig 10:**
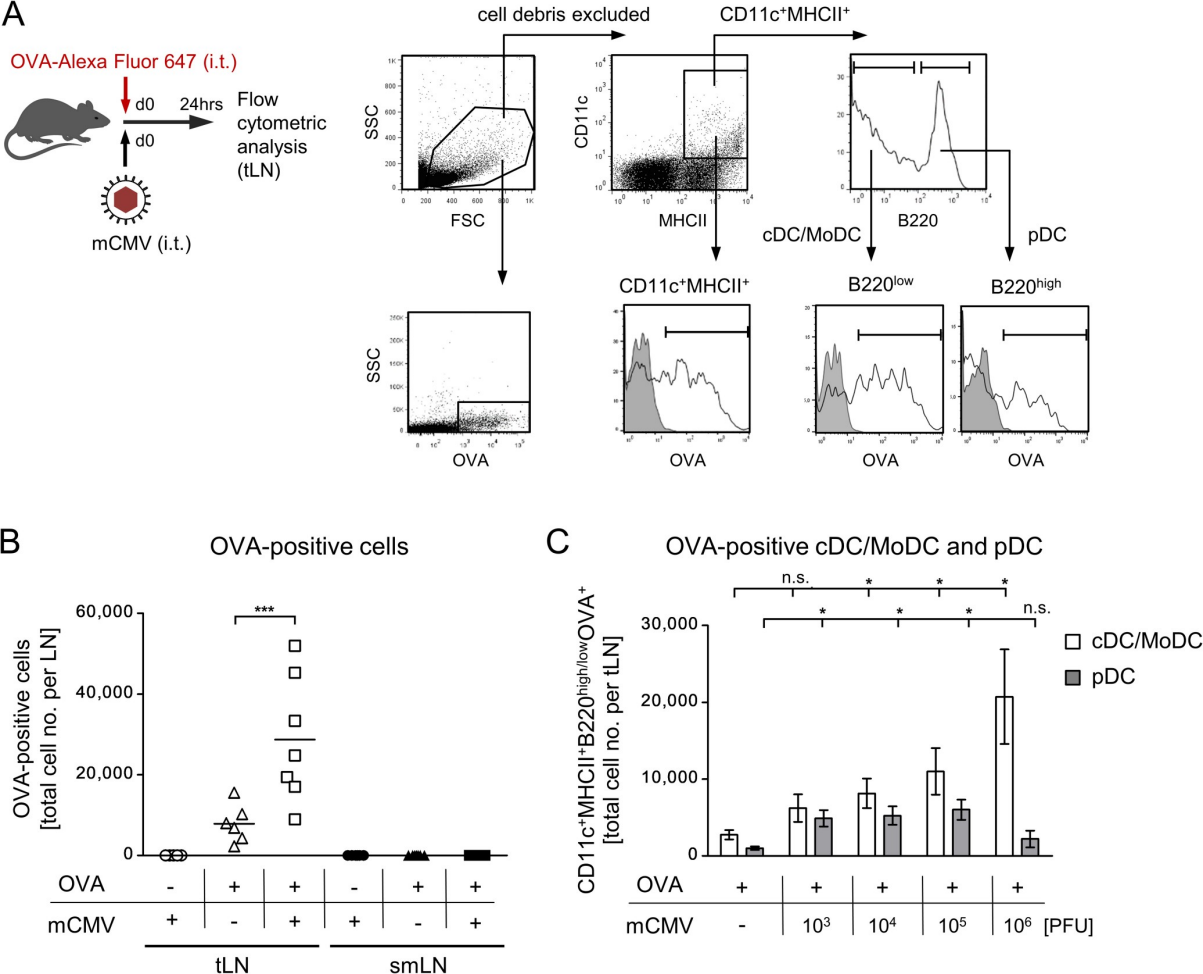
Selective migration of OVA-laden DC subpopulations to the draining tLNs. (A) Experimental design and gating strategy for the identification of major DC subpopulations that take up fluorescent OVA and migrate to the tLNs. An electronic gate was set on viable tLN-derived cells based on the physical properties size and granularity represented by forward scatter (FSC) und sideward scatter (SSC), respectively. A second electronic gate was set on CD11c^+^MHCII^+^ DCs, and cell surface expression of B220 was used to distinguish between B220^low^ cDCs/MoDCs and B220^hi^ pDCs. Uptake of fluorescent OVA by DCs in general as well as by both subpopulations is shown in fluorescence histograms. Shaded histograms represent controls performed with unlabeled OVA. (B) Quantitation of OVA-laden cells that migrated to the draining tLNs or to the non-draining submandibular LN (smLN) in response to the sensitization conditions indicated. Symbols represent individual mice. Mean values are indicated. (C) Quantitation of OVA-laden cDCs/MoDCs and pDCs that migrated to the draining tLN in response to OVA sensitization and co-exposure to graded doses of virus. Bars represent mean values +/- SEM. Shown are data compiled from 2 independent experiments. Asterisk-coded statistical significances: *P≤0.05; ***P≤0.001; n.s.: not significant.

For identifying the DC subset(s) involved in the sensitization for AAD, we set out to first distinguish between a DC population composed of conventional DCs (cDCs) and monocyte-derived DCs (MoDCs) on the one hand, and plasmacytoid DCs (pDCs) on the other. By analyzing the expression of B220 in MHCII^+^CD11c^+^ DCs present in tLNs after combined airway exposure to fluorescent OVA and mCMV (Fig 10), B220^low^ cDCs/MoDCs and the B220^high^ pDCs were found to have taken up the fluorescent OVA (Fig 10A). OVA-laden DCs localized selectively to the draining tLN, and not to the non-draining sub-mandibular LN (smLN) (Fig 10B). Corresponding to the conditions for AAD, significant numbers of OVA-laden DCs were found in the tLNs only after combined airway exposure to OVA and mCMV. Notably, although OVA-laden cDCs/MoDCs and pDCs both migrated to the tLNs, only cDCs/MoDCs migrated in response to mCMV in a virus dose-dependent manner, and predominated in number (Fig 10C). This finding led us to focus on cDCs/MoDCs.

**Fig 11:**
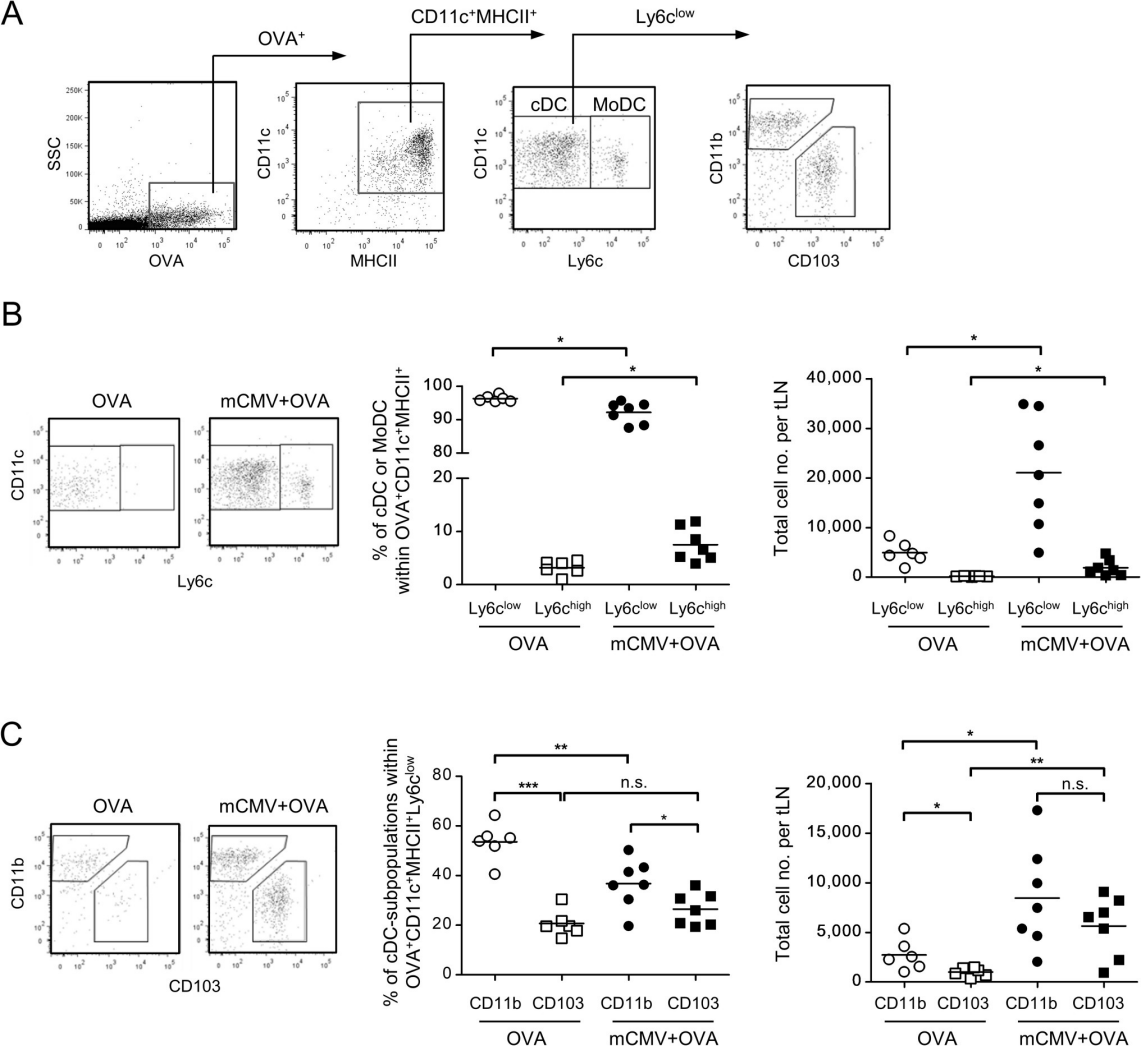
Identification of cDC subsets that take up OVA and migrate to draining tLNs. The experimental design corresponds to that of Figs 9 and 10, using fluorescence-labeled OVA to restrict the analysis to cells that have captured OVA in airway mucosa and migrated to draining tLNs. (A) Gating strategy to distinguish between Ly6c^low^ cDCs and Ly6c^high^ MoDCs, as well as between cDC subsets based on mutually exclusive expression of CD11b and CD103, defining CD11b^+^ cDCs and CD103^+^ cDCs, respectively. (B) Relative and absolute quantitations of OVA-laden Ly6c^low^ cDCs and Ly6c^high^ MoDCs present in tLNs after airway sensitization to OVA alone (OVA) or combined with mCMV (mCMV + OVA). (C) Relative and absolute quantitations of OVA-laden CD11b^+^ cDCs and CD103^+^ cDCs present in tLNs after airway sensitization to OVA alone (OVA) or combined with mCMV (mCMV + OVA). Symbols in (B) and (C) represent data from individual mice with mean values marked. Data were compiled from 2 independent experiments with altogether 6–7 mice per experimental group. Asterisk-coded statistical significances: *P≤0.05; **P≤0.01; ***P≤0.001; n.s.: not significant.

OVA-laden cDCs and MoDCs were distinguished by low and high expression of Ly6c, respectively, and Ly6c^low^ cDCs were subclassified based on mutually exclusive expression of CD11b and CD103 (Fig 11A). Although Ly6c^high^ MoDCs were found to be somewhat elevated in response to mCMV, their relative and absolute numbers are very low compared to cDCs (Fig 11B), so that their contribution to sensitization for AAD is supposedly minor.

Upon OVA sensitization alone, relative and absolute numbers of OVA-laden CD11b^+^ cDCs in tLNs exceed those of CD103^+^ cDCs. Notably, under conditions of co-exposure to mCMV, both subsets equal in significantly increased absolute numbers.

Altogether, the data provide a reasonable argument to conclude that both subsets of migratory cDCs are activated by mCMV in the airway mucosa, take up OVA, and migrate to the draining tLNs for antigen presentation.

## Discussion

Host-to-host transmission of hCMV mostly takes place in early childhood during intimate social contacts of the child with family members or peers who shed the virus with saliva. Local mucosal infection can spread to airway mucosa, where virus and inhaled environmental antigens can meet at airway mucosa-resident DCs capable to migrate upon activation. It was the aim of our study to model this epidemiologically realistic situation for investigating a putative contribution of primary CMV airway infection to sensitization by antigens/allergens that predispose the child for AADs, including asthma. The question is difficult to address by clinical investigation, because primary CMV airway infection in early childhood is usually not diagnosed, so that a link between CMV and AAD years later in life is not obvious to be considered. The here presented data from a mouse model predict that CMV airway infection at the time of exposure to inhaled antigens/allergens can indeed enhance allergenic sensitization that predisposes for AAD. Importantly, the model shows that even a protein antigen that has low-to-no allergenic potential on its own can sensitize for AAD when CMV activates airway mucosa-resident DCs for more efficient antigen uptake. Such a mechanism is medically relevant as it significantly broadens the spectrum of potential environmental inducers of AAD.

The data have consistently shown that histopathological key parameters defining AAD, namely airway inflammation, thickening of airway epithelium, and the lead symptom goblet cell hyperplasia depend on airway co-exposure to a potential allergen and CMV, whereas either exposure alone fails. IgE secretion and IL-5-driven airway eosinophilia have also been associated with AAD (for a review see [63]). While we detected an enhanced IgE secretion in the AAD group *mCMV/OVA//OVA*, eosinophila was not observed despite IL-5 upregulation. This finding is in accordance with work in the mouse model, describing that mCMV infection actually decreases airway eosinophilia [15]. In this context it is important to call attention to a clinical asthma phenotype in humans, known as non-eosinophilic asthma (NEA). NEA is characterized by airway inflammation with absence of eosinophils and represents severe asthma cases, as its most relevant clinical trait is its poor response to standard asthma treatments, especially to inhaled corticosteroids [70]. At the present state, we would not go so far to claim to have here described a murine model for NEA, but the clinical example clearly shows that absence of eosinophilia in our AAD model is certainly no valid argument against the predictive value of this mouse model for AAD entities that exist in humans.

The grade of AAD observed here was not full-blown asthma, as respiratory function was not yet critically affected in the AAD group *mCMV/OVA//OVA*. As a perspective, however, we propose that we observed here a pre-asthmatic stage that may develop into clinical asthma when the recall response is further amplified by additional rounds of challenge exposure. This remains to be tested in future work.

As to the mechanism, the data delineate a causal chain in which CMV activates DCs residing in the airway mucosa, as indicated by expression of CD80 and CD86. This activation is associated with a more efficient uptake of airborne antigens. OVA-laden DCs, in particular migratory CD11b^+^ as well as CD103^+^ cDCs, then specifically migrate to the draining lymph nodes, the tLNs, for presenting OVA peptides to prime naïve T cells. While CD11b^+^ cDCs mediate MHCII-restricted Th2 priming to respiratory allergens/antigens and have been associated with the pathogenesis of asthma [71], CD103^+^ cDCs represent the antigen cross-presenting cDC subset in airway mucosa [72]. Accordingly, also in our model, OVA-laden CD11b^+^ cDCs are likely the inducers of the observed Th2-driven AAD, whereas the activation of CD103^+^ cDCs explains the MHCI-restricted priming of CD8^+^ T cells specific for the OVA-derived antigenic peptide SIINFEKL.

It is established knowledge in the allergy field that Th2-derived IL-9 and IL-4/IL-5 induce goblet cell hyperplasia either directly or indirectly via neutrophilic granulocytes, respectively [60–62]. In addition, IL-25 contributes to AAD by its stimulating effect on IL-4 and IL-5 [63]. The conclusion that viral activation of CD11b^+^ cDCs in airway mucosa is the critical triggering incident in sensitization for AAD is supported by the previous finding that administration of activated *ex vivo* OVA-pulsed DCs to the airways primes for a Th2 response in the lungs and predisposes for pulmonary goblet cell hyperplasia [73].

A missing piece in the chain of evidence is the demonstration of a quantifiable number of Th2 cells that recognize a single, though immunodominant, OVA epitope (OVA_323-339_) in the lungs. This low frequency corresponds to the generally very low response of CD4^+^ T cells in the lungs of mice of the AAD group *mCMV/OVA//OVA*. In this context it is informative to note that in an unrelated mouse model of pulmonary infection with a recombinant influenza virus expressing epitope OVA_323-339_, the frequency of the OVA epitope-specific Th1 cells in the lungs was found to be 10-fold lower than the frequency of CD8^+^ T cells specific for MHCI epitope NP_264-272_ of influenza virus nucleoprotein [74], even though replication of the recombinant virus is expected to result in a particularly efficient priming to OVA.

The quantitatively low OVA-specific Th2 response in the lungs of the AAD group *mCMV/OVA//OVA* contrasts with the production of isotype-switched OVA-specific serum antibodies also observed only in the AAD group, which clearly indicates sufficient activation of the CD4^+^ T-helper cell/B-cell axis, though this is likely to take place in the B-cell areas of lymphoid tissues rather than in lung tissue.

Nonetheless, OVA-specificity of AAD induction is beyond question since AAD required both OVA sensitization and OVA challenge. In addition, an involvement of Th2 cells that reside in the lungs became evident from a CD4^+^ T cell-dependent Th2 cytokine transcription profile in the lungs of *mCMV/OVA//OVA* mice. This included enhanced gene expression of interleukins IL4, IL-5, IL-9, and IL-25, all known to induce goblet cell hyperplasia, the lead histopathological sign of tissue remodeling that defines AAD. The presence of Th2 cells in AAD lungs became evident from the recall response of *in vivo* primed pulmonary Th2 cells by restimulation with full-length OVA protein in cell culture, which led to significant levels of IL-5 specifically in the AAD group *mCMV/OVA//OVA*.

We thus conclude that the *in situ* frequency of OVA peptide-specific Th2 cells is very low in the lungs and that minute numbers of Th2 cells suffice in lung tissue for inducing AAD by local delivery of AAD-driving interleukins IL-4, IL-5, IL-9, and IL-25. IFN-γ-secreting viral epitope-specific Th1 cells as well as CD8^+^ T cells are apparently not critically involved in AAD, as their respective frequencies were about the same in AAD group *mCMV/OVA//OVA* and non-AAD group *mCMV/—//OVA* (Fig 5A).

Notably, UV-inactivated mCMV also induced AAD. In this context it is of interest that non-infectious but enveloped and therefore entry-competent subviral particles of hCMV, the so-called dense bodies, were found to stimulate maturation and activation of immature human DCs [75]. So, apparently, signaling associated with the viral entry process is sufficient to activate DCs. Conformance of mCMV with hCMV in this respect strengthens the validity of the model in predicting activation of DCs in airway mucosa by hCMV. The molecular mechanism by which mCMV activates DCs has been extensively addressed in literature, although studies on mCMV interactions with DC subsets so far focused primarily on pDCs. Despite the fact that OVA-laden cDCs were identified here as the DCs that migrate to draining tLNs for allergenic sensitization, activation by mCMV applies to most DC subsets, including pDCs, as we have found OVA-uptake and CD80/CD86 expression by essentially all MHCII^+^CD11c^+^ DCs. For pDCs it is known that they are activated by mCMV through TLR7 and TLR9 sensing and signaling, leading to the production of type I interferons. Furthermore, all subsets of splenic DCs were found to be activated by mCMV through MyD88 adaptor-dependent TLR9 signaling to produce IL-12 (for reviews, see [76–78]). CD8α^+^cDCs and CD11b^+^cDCs can be infected by mCMV *in vivo*, whereas pDCs are not infectable and nonetheless sense mCMV (reviewed in [76]). All this collectively indicates that productive infection of DCs is not required for mCMV-mediated DC activation that represents the initializing event in the allergenic sensitization phase resulting in AAD.

Pathophysiological interdependence of seemingly unrelated disease entities, resulting in co-morbidity, is an important, though rarely experimentally addressed, issue in medicine. In a sense, our model is a co-morbidity model in that it links CMV infection to AAD. Notably, while we have shown that viral activation of CD11b^+^ cDCs in airway mucosa promotes Th2-driven AAD, a mirror image was recently presented by linking AAD/asthma to influenza virus infection [79]. In that work, prior airway exposure to an inhaled potent allergen activated the CD11b^+^ cDCs in the airway mucosa and their migration into the draining LNs, which unexpectedly resulted in a more efficient priming of protective antiviral CD8^+^ T cells, a function usually attributed in influenza virus infection to the antigen cross-presenting CD103^+^ cDCs [80].

All in all, our data provide reasonable evidence to propose that activation of migratory CD11b^+^ cDCs by mCMV in the airway mucosa to enhanced antigen uptake, migration into the draining tLN, and presentation of allergen epitope(s) to Th2 cells elicits AAD. The medically most important aspect, in our view, is the finding that CMV infection, as predicted by the here presented model, can “convert” a harmless, inhaled protein antigen to an allergen. As a perspective, future work will aim to expand the model for defining conditions under which CMV airway infection could possibly contribute to the development of full-blown asthma, and to address the question if CMV might be involved in the pathogenesis of non-eosinophilic human forms of asthma, NEA.

## Materials and methods

### Mice

Female C57BL/6 mice were purchased from Harlan Laboratories and housed in the translational animal research center (TARC) of the University Medical Center of the Johannes Gutenberg-University Mainz for at least 1 week under specified-pathogen-free (SPF) conditions. Mice were used at the age of 8-to-12-weeks.

### Ethics statement

Animal experiments were approved according to German federal law §8 Abs. 1 TierSchG by the ethics committee of the Landesuntersuchungsamt Rheinland-Pfalz, permission numbers 177-07/G09-1-004 and 177-07/G 14-1-015.

### Intratracheal applications

Intratracheal (i.t.) applications were performed essentially as described [81,82]. In brief, mice were anaesthetized by intraperitoneal (i.p.) injection of ketamin/rompun (Ketamin-ratiopharm, Ratiopharm, Ulm, Germany; Rompun 2%, Bayer, Leverkusen, Germany) and attached on a restraining device. The tongue was carefully pulled aside and with the aid of a cold light positioned at the throat, a crystal tip was placed into the trachea. First, mCMV (cell culture-propagated and purified strain Smith, ATCC VR-194/1981, [83]) was instilled in a volume of 20 μl diluted in PBS. As a number of virological and immunological parameters proved 10^6^ PFU to be the optimal infection dose for i.t. application, experiments were performed with 10^6^ PFU of mCMV if not indicated otherwise. After infection, 20 μl of either OVA-Alexa Fluor 647 conjugate (4mg/ml in PBS; catalog no. O34784, Invitrogen, Germany) or endotoxin-free OVA (EndoGrade Ovalbumin, 4mg/ml in PBS; catalog no. 321001, Hyglos, Bernried, Germany) was administered. PBS served as control. Experimental groups consisted of at least 4 mice.

### Sensitization/challenge protocol and readouts

To induce antigen sensitization via the airways, OVA and mCMV were administered i.t. on day 0 as described above. Subsequently, allergic airway inflammation was provoked by repetitive challenge exposures to OVA (albumin from chicken egg white; catalog no. A5503, Sigma) on days 14, 15, and 16. For this, an OVA solution (1% in PBS) was freshly prepared for every use and nebulized for 20 min with an ultrasonic nebulizer (NE-U17, Omron, Hoofdorp, The Netherlands). After the last challenge, the following parameters were determined for groups of usually 4–5 mice tested either individually (*i-iv*) or as a pool (*v-vii*): (*i*) OVA-specific serum immunoglobulins and IL-5 by ELISA, (*ii*) cellular composition of the broncho-alveolar lavage (BAL) by cytospins, (*iii*) inflammation of the lungs by histology, (*iv*) virus titers, (*v*) epitope-specificity of pulmonary CD4^+^ and CD8^+^ T-cell infiltrates by ELISPOT assay, (*vi*) phenotype of T-cell infiltrates in lung tissue and BAL by cytofluorometry, and (*vii*) gene expression analysis.

Blood samples were collected in tubes, using a 1-ml syringe, and stored at room temperature until sera preparation. BAL was performed as described below and its cellular composition was analyzed following fixation and staining of the cytospins by using a Microscopy Hemacolor-Set (Merck, Darmstadt, Germany). Percentages and absolute numbers were calculated for each cell type. Lung parenchyma was prepared as described below for cytofluorometric analysis of T-cell subsets and for determination of CD4^+^ and CD8^+^ T-cell frequencies by ELISPOT assay. For histological analysis, lungs were fixed and prepared as described below. To obtain cells for cytofluorometric analysis, the left lung lobe was ligated on the afferent bronchus, removed, and stored in PBS on ice until further preparation.

### Depletion of T-cell subsets in vivo

Mice were treated essentially as described in the sensitization/challenge protocol described above. One day before the first challenge (d13), T-cell depletion was performed by i.v. injection of purified monoclonal antibodies directed against CD4 (clone: YTS191.1; 0.8mg/mouse) or CD8 (clone: YTS169.4; 1.3mg/mouse).

### Preparation of single cell suspensions from different tissues

#### Lungs

Mice were lethally anesthetized by carbon dioxide inhalation or by an overdose of Narcoren (Boehringer Ingelheim Vetmedica, Ingelheim, Germany). BAL leucocytes were isolated by performing BAL essentially as described [82], using DPBS + 2% FCS to flush the airways, and erythrocytes were lysed. Mononuclear leucocytes from lung tissue were isolated essentially as described [26,83], with modifications. In brief, after performing BAL, lungs were perfused via the right ventricle to remove circulating cells from the capillary bed of the lungs. Lungs were excised, tracheae, bronchi, and pulmonary LN were discarded, and the lung lobes were minced. Digestion of tissue derived from 3–5 lungs was performed in 15 ml of supplemented DMEM, containing collagenase A (1.6 mg/ml; catalog no. 10 103 586 001, Roche, Mannheim, Germany) and DNase I (50μg/ml; catalog no. DN-25, Sigma, Steinheim, Germany) for 1 h at 37°C with constant stirring. Mononuclear leucocytes were enriched by density gradient centrifugation for 30 min at 760 x g on Lymphocyte Separation Medium 1077 (catalog no. J15-004, PAA, Cölbe, Germany). For isolation of pulmonary mononuclear leucocytes from individual mice [81], perfusion was performed via the right ventricle and excised lungs were transferred into 50-ml tubes. Collagenase type I (0.5 mg/ml; catalog no. C9891, Sigma) was added and after an incubation for 45 min at 37°C in a shaking water bath, cells were passed at least 3 times through a cannula (20G 0.9 mm x 40 mm) in a 10-ml syringe and then through a 70-μm cell strainer to obtain a single cell suspension. Erythrocytes were lysed, and cells were adjusted to 1x10^7^ cells per ml of assay medium.

#### Lymphoid tissues

For preparing single cell suspensions of lymphocytes, spleens were minced and passed through a cell strainer, followed by lysis of erythrocytes. Tracheal LN (tLN) and submandibular LN (smLN) were directly passed through a cell strainer. Cells were washed and viable cells were counted. For cytofluorometric analysis, cells were adjusted to 1x10^7^ cells per ml of assay medium.

### Cytofluorometric analyses

#### Phenotype of T-cell subpopulations

Single cell suspensions were prepared from lung tissue and BAL (see above). Unspecific staining was blocked with unconjugated anti-FcγRII/III antibody (anti-CD16/CD32; clone 2.4G2, BD Biosciences, Heidelberg, Germany), and cells were stained with the following antibodies for multi-color cytofluorometric analyses: PE- and PE-Cy5-conjugated anti-TCRβ (clone H-57-597; BD Biosciences), ECD-conjugated anti-CD8α (clone 53–6.7; Beckmann Coulter, Krefeld, Germany), PE-Cy5-conjugated anti-CD8α (clone 53–6.7; BD Biosciences), FITC-conjugated anti-CD4 (clone GK1.5; BD Biosciences), PE-conjugated anti-KLRG1 (clone 2F1; eBioscience, San Diego, CA), and PE-Cy7-conjugated anti-CD62L (clone MEL-14; Beckman Coulter). Cytofluorometric analyses were performed with flow cytometer LSRII and FACSDiva software (BD Biosciences) or with flow cytometer FC500 and CXP analysis software (Beckman Coulter).

#### DC subpopulations

Single cell suspensions prepared from tissues were adjusted to appropriate cell numbers and incubated with Fc-receptor blocking antibody to prevent unspecific binding (see above). For the analysis of DC subpopulations and their activation state, the following antibodies were used: FITC-conjugated anti-MHCII (clone M5/114.15.2; eBioscience), PE-conjugated anti-CD11c (clone HL3; BD Biosciences), PercP-Cy5.5-conjugated anti-B220 (clone RA3-6B2; BD Biosciences), PE-conjugated anti-CD86 (clone GL1; BD Biosciences), PE-conjugated anti-CD40 (clone 03/23; BD Biosciences), PercP-Cy5.5-conjugated anti-CD197 (CCR7) (clone 4B12; BD Biosciences), PercP-Cy5.5-conjugated anti-CD80 (clone 16-10A1; BD Biosciences), Pe-Cy7-conjugated anti-CD11c (clone HL3, BD Biosciences), PE-conjugated anti-CD103 (clone M290; BD Biosciences), PercP-Cy5.5-conjugated anti-CD11b (clone M1/70; BD Biosciences), and APC-Cy7-conjugated anti-Ly6c (clone AL-21; BD Biosciences). Following incubation, cells were washed and resuspended in fixation buffer (4% PFA).

Flow cytometric measurements were performed on a FACSCanto II (BD Bioscience) using Diva software. Analysis of raw data and graphics were generated using FlowJo software (Tree Star Inc., Ashland, OR). The total numbers of cells of interest were calculated by allocating the percentages determined by cytofluometric cell phenotyping to the yield of total cells per organ.

### ELISA

#### Quantitation of serum immunoglobulins

Serum was collected 48 hrs following the last OVA challenge. OVA-specific IgG1-, IgG2b- and IgG2c titers were determined by ELISA following the manufacturer`s protocol (Biotin rat α-IgG1: clone A85-1, BD Biosciences; Biotin rat α-IgG2b: clone R12-3, BD Biosciences; Biotin goat α-IgG2c, Biozol, Eiching, Germany). OVA-specific IgE titers were analyzed by ELISA (α-IgE; clone: EM95.3.1, donated by Dr. E. Schmitt, Institute of Immunology, University Medical Center Mainz, Germany) as described previously [84]. Antibody titers were defined as the reciprocal serum dilution that resulted in an OD = 0.2 based on linear regression analysis.

#### Quantitation of Th2 lymphokine IL-5

Single cell suspensions from the lungs were prepared as described above, and 5 x 10^5^ cells were seeded in 0.1 ml cell cultures with 25 μg of OVA (Sigma, GradeV) in IMDM medium (10% FCS, 1% penicillin/streptomycin), followed by 72 hrs of incubation at 37°C. IL-5 in the supernatants of the OVA-restimulated cells was quantitated by an IL-5 ELISA (R&D Systems, Minneapolis, MN).

### Histology

Lungs were fixed by inflation (1 ml) and immersion in 4% formalin, followed by embedding in paraffin. Tissue sections were prepared and stained with Hematoxylin/Eosin (HE) to analyse tissue inflammation, or with a combined Periodic Acid Schiff (PAS)/HE staining to identify mucus-producing goblet cells.

Airway inflammation was scored semi-quantitatively on HE slides. For this, five randomly selected areas were scored by two experienced observers blinded to experimental groups. Inflammation was scored on a scale from 0 to 4. The score is based on the following inflammatory conditions: {0}, all airways and vessels are free of inflammatory infiltrates. {1}, some infiltrates are detectable around airways and vessels, and are up to two layers in thickness. {2}, the majority of airways and vessels show infiltrates with a thickness of up to two layers, only occasionally more layers can be found. {3}, the majority of airways and vessels show inflammatory infiltrates. Many layers are thicker than two layers, ranging from 3–8 layers. {4}, all airways and vessels are surrounded by thick layers of inflammatory cells.

PAS-positive goblet cells were quantified per millimeter of basal membrane on at least three different representative airways on PAS-stained slides. Airway thickness was measured on three independent HE stained slides, with at least three different representative airways per slide, so that at least 9 airways were evaluated. The measurements were performed by using an imaging software (analySIS, Soft Imaging Systems, Stuttgart, Germany).

### Quantitation of infectious virus

Infectious virus (expressed as plaque forming units, PFU) was quantitated for whole organ homogenates by a virus plaque assay performed on monolayers of mouse embryo fibroblasts, making use of increasing the sensitivity by the method of centrifugal enhancement of infectivity ([83] and references therein).

### Peptides

Custom peptide synthesis to a purity of > 80% was performed by JPT Peptide Technologies (Berlin, Germany). Synthetic peptides were used for exogenous loading of C57BL/6 (H-2^b^) spleen cells and of EL-4 (H-2^b^) cells with panels of MHCII- and MHCI-presented peptides of mCMV or OVA, respectively. Peptide-laden cells were used as stimulator cells in the ELISPOT assay, as described below.

Peptides recognized by CD4^+^ T cells: m18 (NERAKSPAAMTAEDE) [52], M25 (LYETPISATAMVIDI) [53], m139 (TRPYRYPRVCDASLS) [52], m141 (LVVFSDPNADAATSV) [52], and m142 (RYLTAAAVTAVLQDF) [53], and OVA_323-339_ (ISQAVHAAHAEINEAGR) [54].

Peptides recognized by CD8^+^ T cells: M38 (SSPPMFRVP), M45 (HGIRNASFI), M57 (SCLEFWQRV), IE3 (RALEYKNL), m139 (TVYGFCLL), m141 (VIDAFSRL), and m164 (GTTDFLWM) [55], and OVA_257-264_ (SIINFEKL) [85].

### ELISPOT assays

Frequencies of virus- and OVA-specific CD8^+^ T cells and CD4^+^ Th1 cells were determined by an IFN-γ-based ELISPOT assay as described ([86,87] and references therein). In brief, graded numbers of immunomagnetically purified CD4^+^ and CD8^+^ T cells from lung tissue of 4–5 mice per group were stimulated for IFN-γ secretion in triplicate assay cultures. C57BL/6 spleen cells and EL-4 lymphoma cells were used as APCs exogenously loaded with synthetic peptides at loading concentrations of 10^−6^ M (CD4^+^ T-cell epitopes) and 10^−7^ M (CD8^+^ T-cell epitopes), respectively.

Frequencies of IL-4 producing CD4^+^ Th2 cells were determined by using the IFN-γ/IL-4 double-color ELISPOT assay, according to the manufacturer’s protocol (CTL, Shaker Hights, OH). The total number of CD4^+^ Th2 cells capable of producing IL-4 upon polyclonal stimulation was determined by the CD3ε-redirected ELISPOT assay ([83] and references therein), using 145-2C11 hybridoma cells as stimulator cells that produce agonistic monoclonal antibody directed against the CD3ε molecule of the T cell-receptor-CD3 signaling complex.

Frequencies of IFN-γ and IL-4 secreting, spot-forming cells and the corresponding 95% confidence intervals were calculated by intercept-free linear regression analysis [86,87]. Spots were counted automatically based on standardized criteria using ImmunoSpot S4 Pro Analyzer (CTL, Shaker Hights) and CTL ImmunoSpot software V5.1.36 Professional DC.

### RNA extraction and gene expression analysis

Total lung RNA was purified from lung tissue from a pool of 5 mice for each group by the RNeasy Micro Kit (Qiagen, Hilden, Germany) with on-column DNase I treatment according to manufacturer’s instructions (Qiagen). 1μg of high-quality total RNA was reverse transcribed using the First Strand Synthesis Kit (Qiagen) to generate the template for the RT^2^ Profiler PCR Array Mouse Th1&Th2 Responses (Qiagen). The PCR reaction was run on an ABI 7500 Real Time PCR System (ThermoFisher Scientific, Darmstadt, Germany) and subsequently analyzed with the SDS Software 1.4.25 following the provided instructions (Qiagen). The CT cut-off was set to cycle 35. The web-based Data Analysis Center provided by the manufacturer was used to generate comparative heat maps and scatter plots, and fold-change was calculated by determining the ratio of mRNA levels to control values using the ΔΔCt method. All data were normalized to an average of four housekeeping genes, *Gusb*, *Hsp90ab1*, *Gapdh*, and *Actb*. A list of analyzed Th1&Th2 related genes is provided online by the manufacturer.

### Statistical analysis

For comparing data from more than two experimental groups, ANOVA was performed with additional Bonferroni correction to determine significant differences between the experimental groups. P values between two groups of interest were calculated by using the unpaired two-tailed Student’s t test with Welch’s correction of unequal variances. Differences between data sets are considered statistically significant (*) for P ≤ 0.05, very significant (**) for P ≤ 0.01, and highly significant (***) for P ≤ 0.001. All analyses were performed with Graphpad Prism 6.04 (GraphPad Software, San Diego, CA).

## Supporting information

S1 Fig Inflammatory cell infiltration of airway epithelia depends on challenge exposure to allergen. (A) Experimental design of sensitization by airway co-exposure to OVA and mCMV, followed by three consecutive challenge exposures to aerolized OVA as the test variable in this experiment. (B) Absolute cell numbers retrieved from airway epithelia by BAL (left three panels) and inflammation score in lung tissue sections (right panel). Symbols represent individual mice of groups that received OVA challenge (OVA, filled diamonds) or were left without OVA challenge (PBS, empty diamonds). Mean values are indicated. Mac, macrophages; Lympho, lymphocytes; Neutro, neutrophilic granulocytes.
(TIF)

S2 Fig Viral infectivity is not required for sensitization. (A) Experimental design of sensitization by airway co-exposure to OVA and to either infectious mCMV (mCMV) or mCMV made replication-incompetent by UV-irradiation (mCMV_UV_), followed by three consecutive challenge exposures to aerolized OVA. (B) Absolute cell numbers retrieved from airway epithelia by BAL (left three panels) and inflammation score in lung tissue sections (right panel). Symbols represent individual mice of groups sensitized by OVA in the presence of infectious mCMV (mCMV, filled diamonds) or in the presence of UV-inactivated mCMV (mCMV_UV_, empty diamonds). Mean values are indicated. Mac, macrophages; Lympho, lymphocytes; Neutro, neutrophilic granulocytes.
(TIF)

S3 Fig Phenotypes of T lymphocytes retrieved from airway epithelia by BAL. Cytofluorometric analysis of BAL-derived T lymphocytes corresponding to the analysis of T lymphocytes dissociated from lung tissue by enzymatic digestion (Fig 4A). For the code of experimental groups, see the legend to Fig 4 and Table 1. Note that group*—/—//OVA* is missing because of a too low yield of infiltrate cells.
(TIF)

S4 Fig Low frequency of OVA epitope-specific IL-4-secreting Th2 cells in lung tissue. Experimental design as outlined and explained in Fig 1A and Table 1, experimental group *mCMV/OVA//OVA*. Frequencies of epitope-specific cells among immunomagnetically-purified CD4^+^ T cells were determined by an IL-4-based ELISPOT assay after stimulation with the synthetic antigenic peptides indicated. For a positive intra-assay control, cells of the same preparation were activated polyclonally through ligation of the CD3ε component of the TCR-CD3 complex with monoclonal anti-CD3ε antibodies (αCD3). Bars represent most probable numbers calculated by intercept-free linear regression analysis of data from graded numbers of effector cells each tested in triplicate cultures. Error bars indicate the 95% confidence intervals.
(TIF)

S5 Fig Expression of Th1/Th2-related genes in lung tissue regulated in response to allergen re-exposure. Shown are the original scatter plots corresponding to Fig 7. (A) Genes regulated relative to reference group*—//—*by OVA challenge in lungs that contain CD4^+^ T cells (group*—//OVA*). (B) Genes regulated relative to reference group*—//—*by OVA challenge in lungs depleted of CD4^+^ T-cells on the day before the first OVA challenge exposure (group *αCD4//OVA*). Up- or downregulation of a specific mRNA was defined by a 1.4-fold change after normalization to housekeeping genes (dashed lines). Data sets were compiled from a pool of 5 biological replicates each. Each dot represents an mRNA species and the corresponding gene.
(TIF)
