# Supplementary figures and images for: Coincident airway exposure to low-potency allergen and cytomegalovirus sensitizes for allergic airway disease by viral activation of migratory dendritic cells
Source: PLoS Pathog. 2019 Mar 7;15(3):e1007595. doi: 10.1371/journal.ppat.1007595 (PMC6405056; doi:10.1371/journal.ppat.1007595)

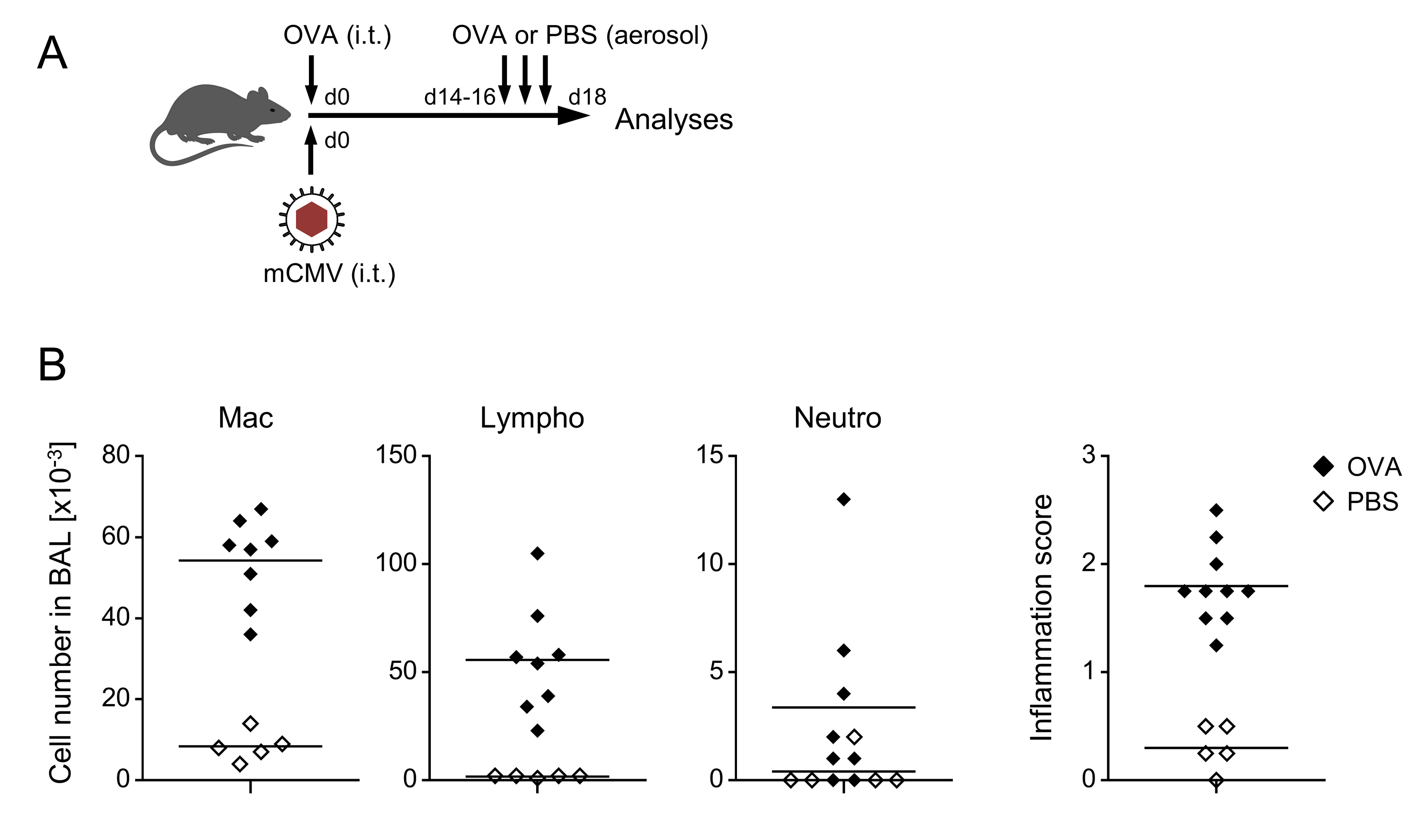

Supplement: S1 Fig — (A) Experimental design of sensitization by airway co-exposure to OVA and mCMV, followed by three consecutive challenge exposures to aerolized OVA as the test variable in this experiment. (B) Absolute cell numbers retrieved from airway epithelia by BAL (left three panels) and inflammation score in lung tissue sections (right panel). Symbols represent individual mice of groups that received OVA challenge (OVA, filled diamonds) or were left without OVA challenge (PBS, empty diamonds). Mean values are indicated. Mac, macrophages; Lympho, lymphocytes; Neutro, neutrophilic granulocytes. (TIF) [file ppat.1007595.s001.tif]

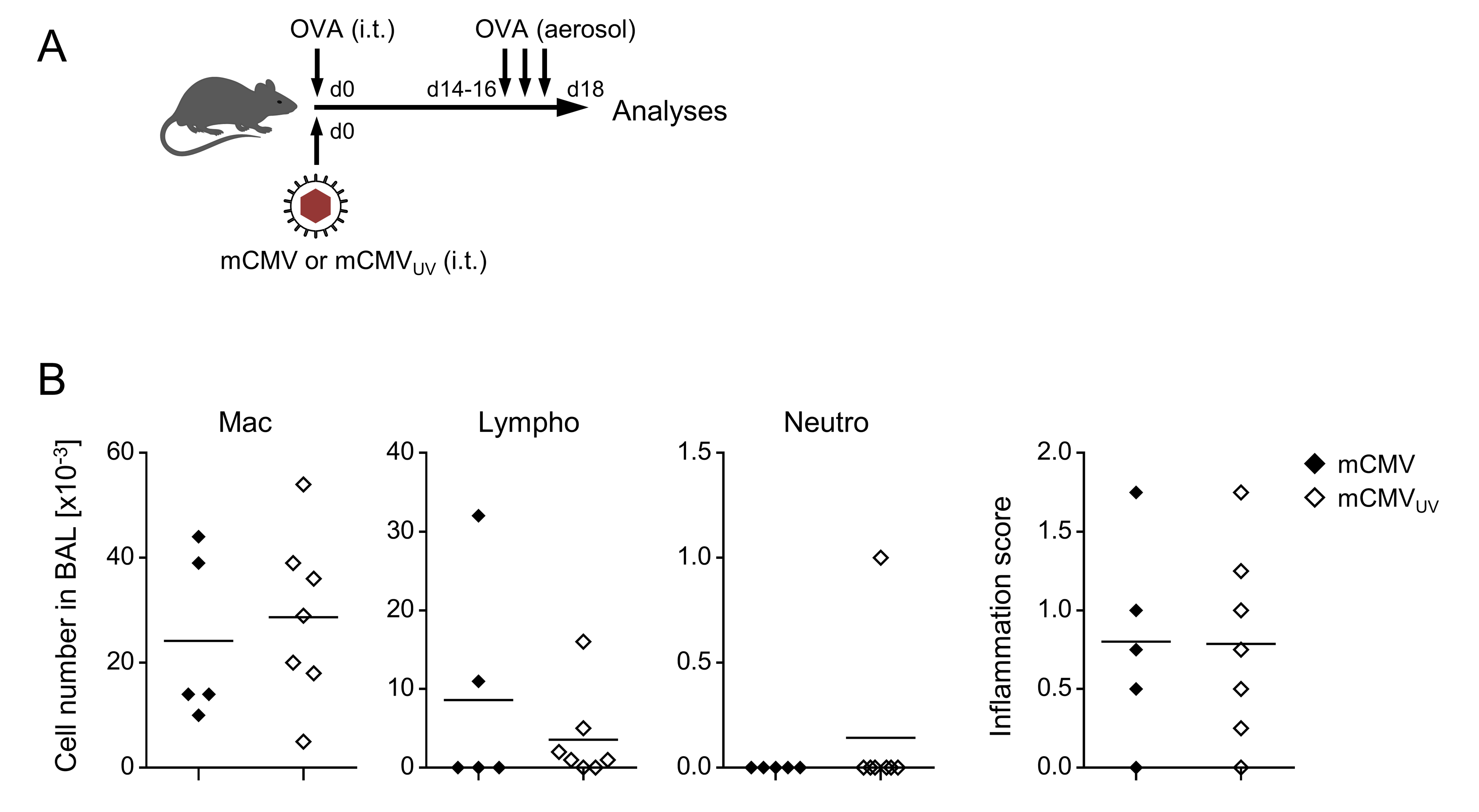

Supplement: S2 Fig — (A) Experimental design of sensitization by airway co-exposure to OVA and to either infectious mCMV (mCMV) or mCMV made replication-incompetent by UV-irradiation (mCMVUV), followed by three consecutive challenge exposures to aerolized OVA. (B) Absolute cell numbers retrieved from airway epithelia by BAL (left three panels) and inflammation score in lung tissue sections (right panel). Symbols represent individual mice of groups sensitized by OVA in the presence of infectious mCMV (mCMV, filled diamonds) or in the presence of UV-inactivated mCMV (mCMVUV, empty diamonds). Mean values are indicated. Mac, macrophages; Lympho, lymphocytes; Neutro, neutrophilic granulocytes. (TIF) [file ppat.1007595.s002.tif]

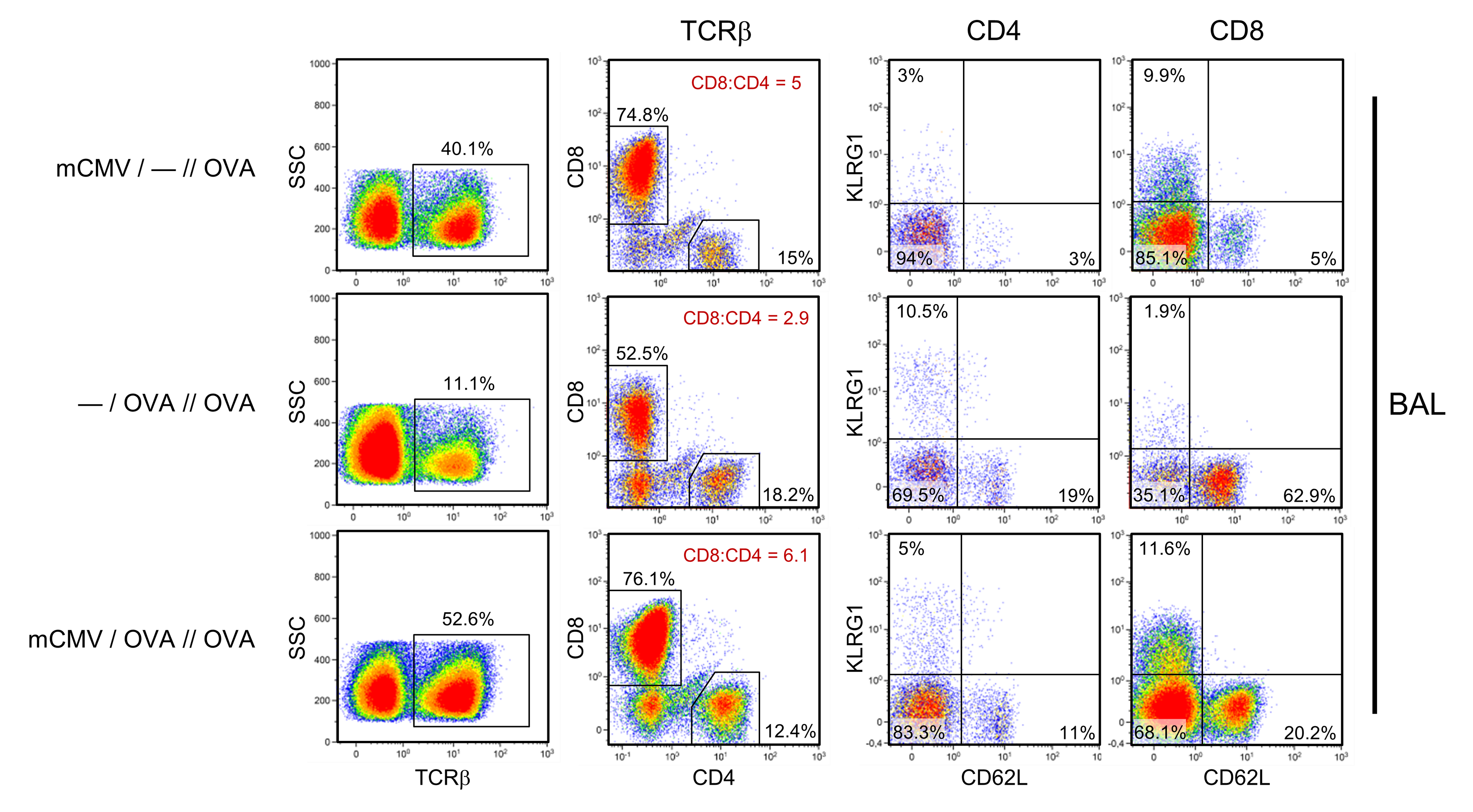

Supplement: S3 Fig — Cytofluorometric analysis of BAL-derived T lymphocytes corresponding to the analysis of T lymphocytes dissociated from lung tissue by enzymatic digestion (Fig 4A). For the code of experimental groups, see the legend to Fig 4 and Table 1. Note that group—/—//OVA is missing because of a too low yield of infiltrate cells. (TIF) [file ppat.1007595.s003.tif]

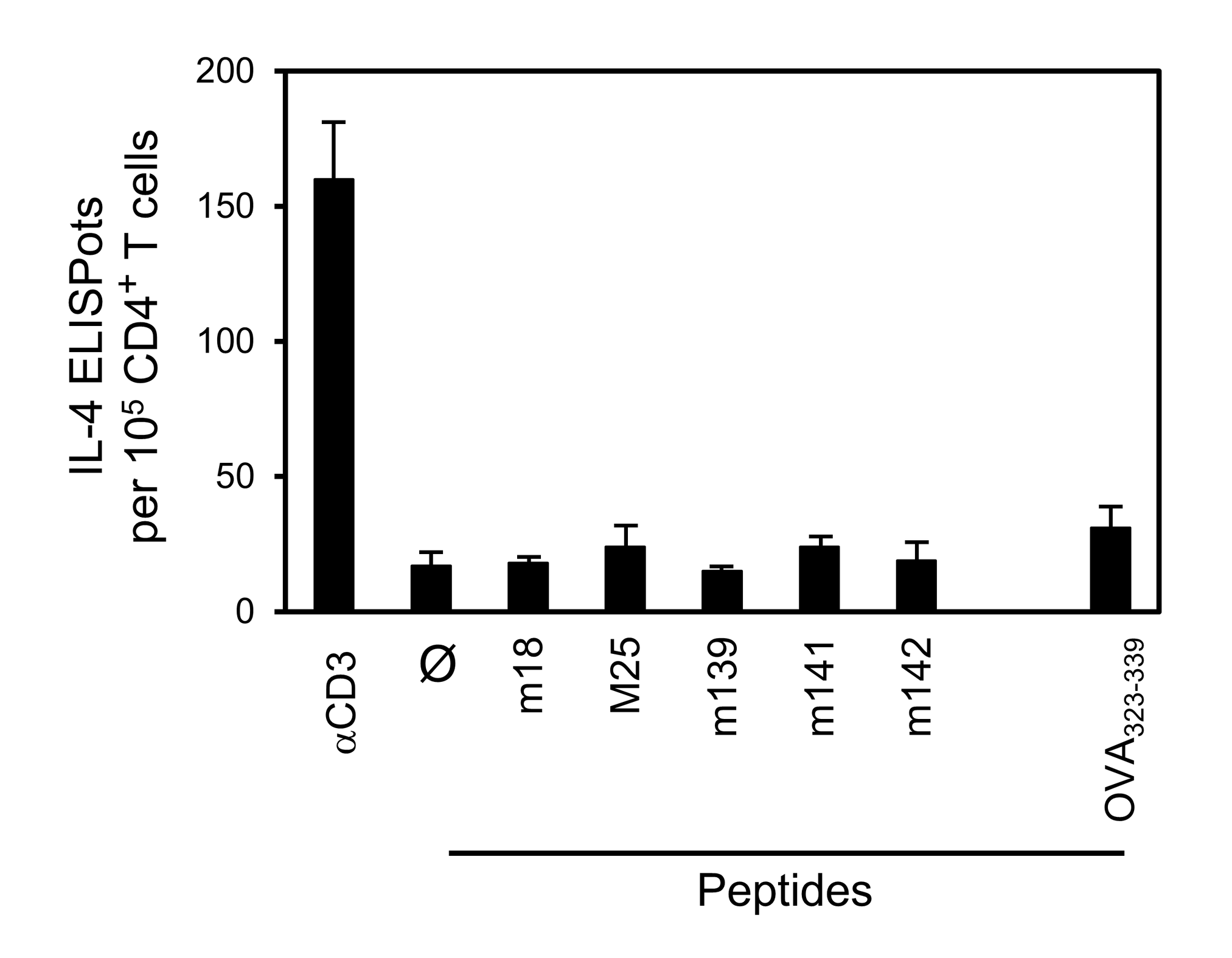

Supplement: S4 Fig — Experimental design as outlined and explained in Fig 1A and Table 1, experimental group mCMV/OVA//OVA. Frequencies of epitope-specific cells among immunomagnetically-purified CD4+ T cells were determined by an IL-4-based ELISPOT assay after stimulation with the synthetic antigenic peptides indicated. For a positive intra-assay control, cells of the same preparation were activated polyclonally through ligation of the CD3ε component of the TCR-CD3 complex with monoclonal anti-CD3ε antibodies (αCD3). Bars represent most probable numbers calculated by intercept-free linear regression analysis of data from graded numbers of effector cells each tested in triplicate cultures. Error bars indicate the 95% confidence intervals. (TIF) [file ppat.1007595.s004.tif]

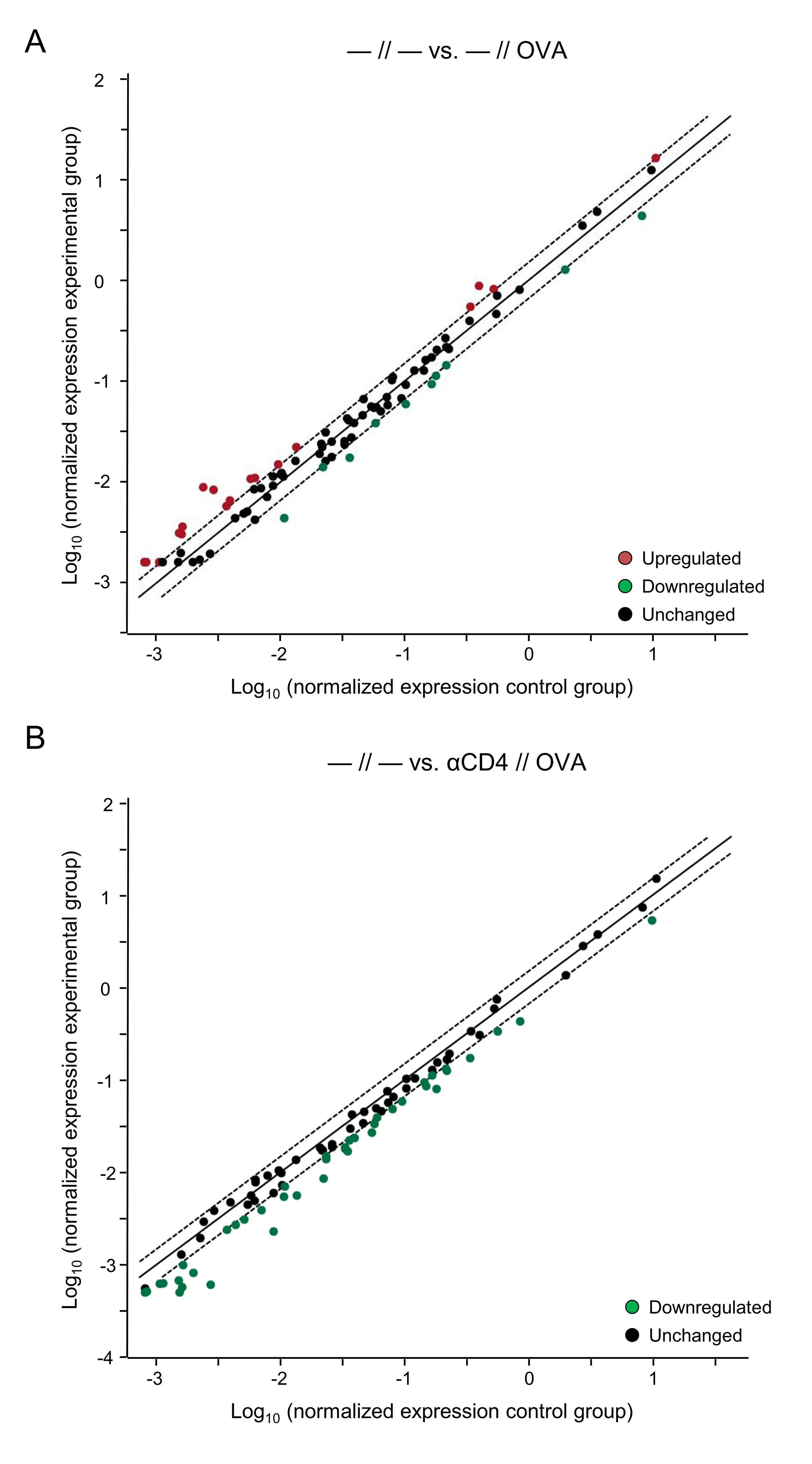

Supplement: S5 Fig — Shown are the original scatter plots corresponding to Fig 7. (A) Genes regulated relative to reference group—//—by OVA challenge in lungs that contain CD4+ T cells (group—//OVA). (B) Genes regulated relative to reference group—//—by OVA challenge in lungs depleted of CD4+ T-cells on the day before the first OVA challenge exposure (group αCD4//OVA). Up- or downregulation of a specific mRNA was defined by a 1.4-fold change after normalization to housekeeping genes (dashed lines). Data sets were compiled from a pool of 5 biological replicates each. Each dot represents an mRNA species and the corresponding gene. (TIF) [file ppat.1007595.s005.tif]
